# STING nuclear partners contribute to innate immune signaling responses

**DOI:** 10.1016/j.isci.2021.103055

**Published:** 2021-08-28

**Authors:** Charles R. Dixon, Poonam Malik, Jose I. de las Heras, Natalia Saiz-Ros, Flavia de Lima Alves, Mark Tingey, Eleanor Gaunt, A. Christine Richardson, David A. Kelly, Martin W. Goldberg, Greg J. Towers, Weidong Yang, Juri Rappsilber, Paul Digard, Eric C. Schirmer

**Affiliations:** 1 Institute of Cell Biology, University of Edinburgh, Kings Buildings, Swann 5.22, Mayfield Road, Edinburgh EH9 3BF, UK; 2 Department of Biology, Temple University, Philadelphia 19121, USA; 3 Division of Infection and Immunity, Roslin Institute, University of Edinburgh, Edinburgh EH25 9RG, UK; 4 School of Biological and Biomedical Sciences, Durham University, Durham DH1 3LE, UK; 5 Department of Infection and Immunity, University College London, London WC1E 6BT, UK; 6 Department of Bioanalytics, Institute of Biotechnology, Technische Universitat Berlin, 13355 Berlin, Germany

**Keywords:** Molecular physiology, Immunology, Virology, Cell biology

## Abstract

STimulator of INterferon Genes (STING) is an adaptor for cytoplasmic DNA sensing by cGAMP/cGAS that helps trigger innate immune responses (IIRs). Although STING is mostly localized in the ER, we find a separate inner nuclear membrane pool of STING that increases mobility and redistributes to the outer nuclear membrane upon IIR stimulation by transfected dsDNA or dsRNA mimic poly(I:C). Immunoprecipitation of STING from isolated nuclear envelopes coupled with mass spectrometry revealed a distinct nuclear envelope-STING proteome consisting of known nuclear membrane proteins and enriched in DNA- and RNA-binding proteins. Seventeen of these nuclear envelope STING partners are known to bind direct interactors of IRF3/7 transcription factors, and testing a subset of these revealed STING partners SYNCRIP, MEN1, DDX5, snRNP70, RPS27a, and AATF as novel modulators of dsDNA-triggered IIRs. Moreover, we find that SYNCRIP is a novel antagonist of the RNA virus, influenza A, potentially shedding light on reports of STING inhibition of RNA viruses.

## Highlights

• A nuclear envelope pool of STING redistributes upon innate immune response activation
• STING has a unique set of nuclear partners that contribute to innate immune responses
• RNA- and DNA-binding STING partners may promote IRF3 transcription factor activation
• STING nuclear partners may explain its protection against RNA viruses

## Graphical abstract

**Figure:**
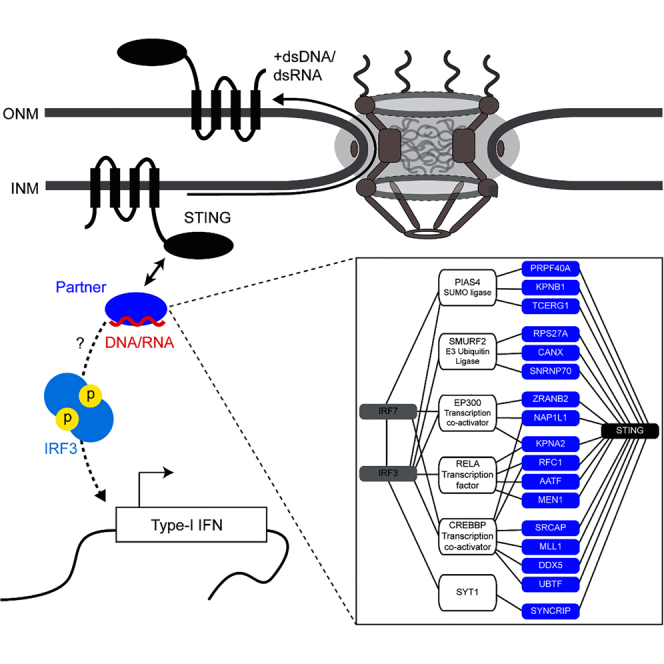

## Introduction

STING (STimulator of INterferon Genes), also called MITA, ERIS, MPYS, NET23, and TMEM173, is an important player in the innate immune response (IIR), the first line of defense against pathogens (Chen and Jiang, 2013; Unterholzner, 2013), yet several studies indicate it has a much wider range of important cellular functions. Its best characterized role is as the essential adaptor protein in innate immune signaling cascades triggered by cytosolic DNA. Cyclic GMP-AMP synthase (cGAS) senses cytoplasmic dsDNA and catalyzes the synthesis of a second messenger, cGAMP, which binds to and activates dimeric STING at the ER, activating IIR signaling cascades that stimulate IRF3/7 transcription factors to activate IIR genes such as type I interferons (IFNs) (Ablasser and Chen, 2019; Ahn and Barber, 2019; Cai et al., 2014; Ishikawa and Barber, 2008; Ishikawa et al., 2009; Motwani et al., 2019; Sun et al., 2013). Although STING does not directly bind to RNA, the replication of multiple positive- and negative-sense RNA viruses is enhanced in the absence of STING (Aguirre et al., 2012; Ding et al., 2018; Franz et al., 2018; Holm et al., 2016; Ishikawa and Barber, 2008; Ishikawa et al., 2009; Maringer and Fernandez-Sesma, 2014; Nazmi et al., 2012; Nitta et al., 2013; Ran et al., 2014; Sun et al., 2009; Yi et al., 2015; Zhong et al., 2008); however, its mechanism for restricting RNA viruses remains to be fully elucidated. Several reports have argued that STING is not involved in interferon activation in response to foreign RNA (Franz et al., 2018; Ishikawa et al., 2009; Li et al., 2013b), so how it acts against RNA viruses is less clear than its counteraction of DNA viruses through type I IFN induction. Other IIR roles for STING have been identified in proapoptotic signaling with MHC II from the plasma membrane (Jin et al., 2008), the induction of autophagy (Gui et al., 2019), and in NF-κB activation downstream of DNA damage (Dunphy et al., 2018). The many distinct functions and localizations reported for STING in IIRs make it difficult to distinguish direct from downstream signaling effects.

Thus far, all these roles have been presumed to occur in the cytoplasm, but the recent finding that STING partner cGAS is also present in the nucleus (Gentili et al., 2019; Jiang et al., 2019; Liu et al., 2018; Volkman et al., 2019; Zierhut et al., 2019) raises the exciting possibility of STING also functioning in the nucleus. This is particularly likely because STING was found in a proteomics study of the nuclear envelope (NE) (Schirmer et al., 2003) and its NE localization depended in part on lamin A (Malik et al., 2010), suggesting it was in the inner nuclear membrane (INM). A subsequent study showing a role for STING in promoting chromatin compaction further suggested a function inside the nucleus (Malik et al., 2014).

The finding of cGAS, STING’s upstream partner in cytoplasmic dsDNA sensing, in the nucleus (Gentili et al., 2019; Jiang et al., 2019; Liu et al., 2018; Volkman et al., 2019; Zierhut et al., 2019) is extremely important. cGAS directly binds DNA, and so a long-standing question in the field was how it was prevented from binding and being activated by chromosomal DNA. Recent studies have shown that in fact a large portion of cGAS is in the nucleus bound to chromosomes (Ablasser and Chen, 2019; Gentili et al., 2019; Ng et al., 2018; Zierhut et al., 2019), so it is critical to keep this pool from activating IIRs. At the same time, this nuclear pool is thought to function in DNA damage responses and tumorigenesis and the cGAS/STING pathway has been linked to an interferon response associated with replication stress owing to the nuclear lamin progerin variant (Kreienkamp et al., 2018). A pool of STING in the nucleus also raises the possibility of nuclear cGAS/STING sensing pathogen nucleic acids inside the nucleus as well as in the cytoplasm and thus activating IIRs from inside the nucleus. It is also possible that nuclear STING could sense RNA viruses that replicate in the nucleus through other partners.

Here we confirm INM residence for endogenous STING and show that INM STING-GFP redistributes from the nucleus to the ER upon treatment with dsDNA or, surprisingly, the dsRNA mimetic poly(I:C). We further show that STING mobility in the NE increases with both DNA- and RNA-triggered immune responses. Moreover, we identify partners of NE localized STING, which are enriched for RNA- and DNA-binding proteins, and testing several of these partners indicates that they can contribute to IIR activation. Importantly, one of the partners identified, SYNCRIP, can protect against infection with the RNA virus, influenza A.

## Results

### STING targets to the INM

**Figure 1:**
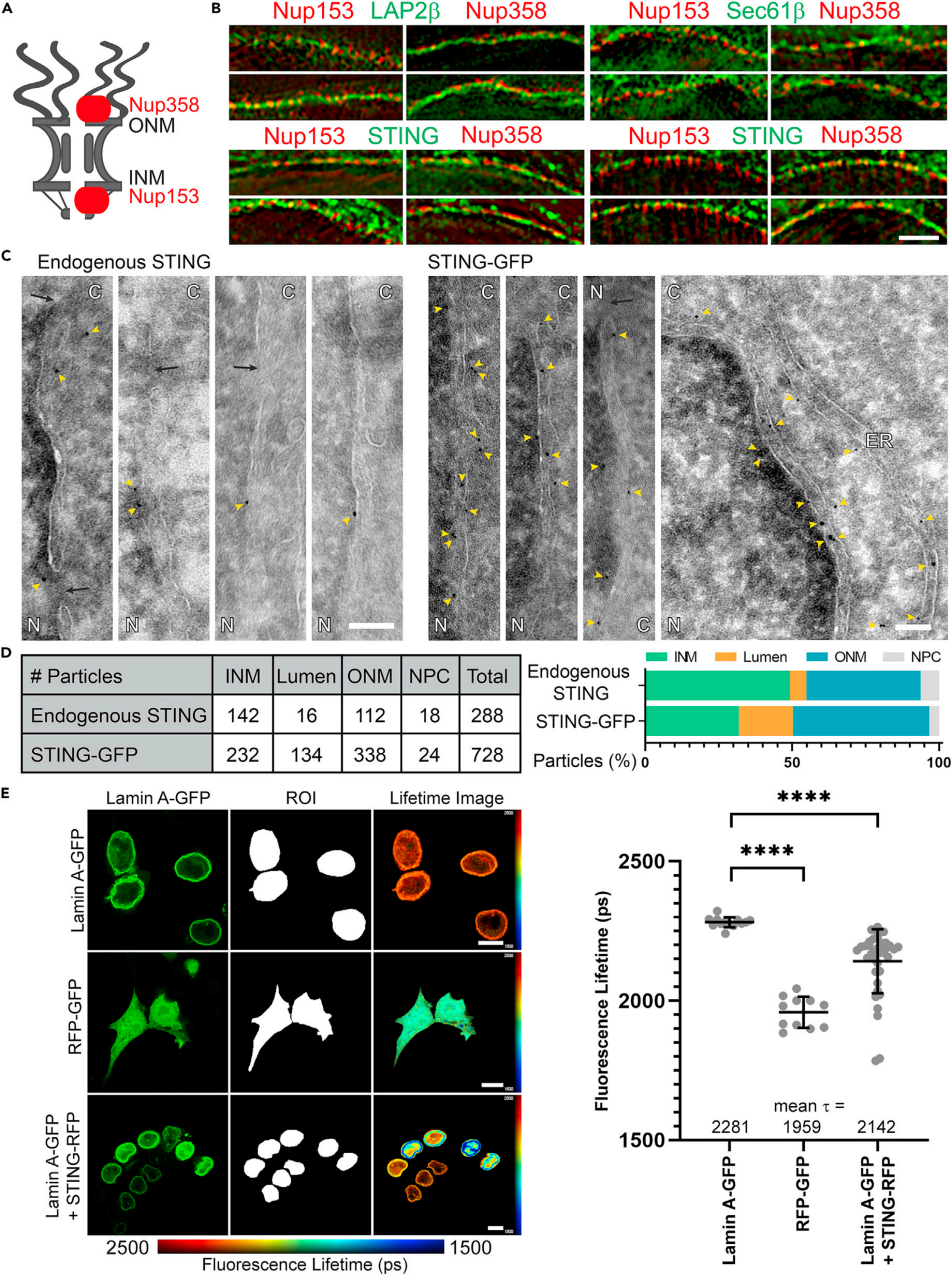
STING inner nuclear membrane localization (A) Schematic of nuclear pore complex (NPC) indicating the location of Nup358 and Nup153 in the NPC. (B) With structured illumination super-resolution (OMX) microscopy, proteins lining up in the same plane as Nup153 indicate localization in the inner nuclear membrane, while proteins lining up in the same plane as Nup358 indicate localization in the outer nuclear membrane. Upper panel controls: LAP2β is known to be in the inner and Sec61β in the outer nuclear membrane. Lower panels: STING is in the inner nuclear membrane in some cells and in the outer nuclear membrane in others. Scale bar, 5 μm. (C) Immunogold electron microscopy for endogenous STING confirms its inner nuclear membrane localization (endogenous STING panels, antibody specificity confirmed in Figures S1 and S2) with particles also observed in the outer nuclear membrane and ER. A much higher number of particles could be observed per image for exogenously expressed STING tagged with GFP that yielded a similar distribution. N, nucleus; C, cytoplasm; yellow arrowheads, immunogold particles; black arrows, NPCs; scale bar, 100 nm. (D) Quantification of the larger volume of data represented in Figure 1C. The apparent increase of particles in the NE lumen for STING-GFP likely reflects enhancement of sectioning artifacts due to the size of the tag. (E) FRET-FLIM indicates an interaction between STING and the lamin A polymer that lines the inner nuclear membrane. Representative images are shown for the lamin A-GFP alone (negative control), a tandem GFP-RFP construct (positive control), and the lamin A-GFP:STING-RFP pairing. Blue indicates a reduction in GFP fluorescence lifetime due to the transfer of photons to the acceptor RFP molecules. Quantification of averaged τ values for the fluorescence lifetime of the donor GFP signal in picoseconds revealed a significant transfer of energy from lamin A to STING, indicative of their interacting. Mean τ values shown ± standard deviation. Ordinary one-way ANOVA with Dunnett’s multiple comparison test, ∗∗∗∗p ≤ 0.0001. Scale bar, 10 μm.

An earlier attempt to determine if the NE pool of STING was in the outer nuclear membreane (ONM) or INM was inconclusive (Malik et al., 2010). Therefore, we used structured illumination (OMX) super-resolution microscopy that can distinguish INM proteins from ONM proteins by their being in the same plane with nuclear basket or cytoplasmic filament proteins of the nuclear pore complexes (NPCs), respectively, that are separated by ∼100 nm (Figure 1A) (Schermelleh et al., 2008). Analyzing several cells on the same coverslip revealed STING-GFP to be in the ONM of some cells and the INM of other cells (Figure 1B). This finding was striking because most NE transmembrane proteins (NETs) tested by this method were unambiguously resident in either the INM or ONM (Korfali et al., 2010; Wilkie et al., 2011). This raised the possibility that STING might redistribute into different NE locations under certain cell-specific conditions.

As the super-resolution approach used STING fused to GFP, we also tested for an endogenous STING NE pool by immunogold electron microscopy (EM) (Figure 1C, left images). Similar numbers of gold particles were observed at the INM (142) as at the ONM (112). Specificity of immunogold labeling was confirmed by the absence of gold particles in samples stained only with secondary antibodies (Figure S1A). To test if the fusion of GFP to STING altered its distribution, a line of HT1080 cells stably expressing STING-GFP was generated and analyzed by immunogold EM (Figure 1C, right images). The GFP tag increased the number of particles that appeared to be in the NE lumen, likely a sectioning artifact for both INM and ONM STING due to the increased distance between gold particle and protein when staining for the GFP tag; nonetheless, there were still a large proportion of clearly identified INM particles (Figure 1D).

Finally, we confirmed the INM pool of STING by a third approach, the ability of C-terminally tagged STING-RFP to accept photons from lamin A-GFP through Förster resonance energy transfer by fluorescence lifetime imaging (FRET-FLIM). The lifetime of the activated lamin A-GFP fluorescence was reduced when cells also expressed STING-RFP as a photon acceptor (Figure 1E). On average, this INM pool of STING reduced the mean lifetime (τ) of lamin A-GFP from 2.281 to 2.142 ns. Expected STING-RFP targeting to the ER/NE was confirmed by confocal microscopy (Figure S1B).

### STING dynamics are altered upon stimulation of IIRs

One possible explanation for STING-GFP being in the INM of some cells but not in others (Figure 1B) is that its different pools might be altered by activation of IIRs, especially because plasmid DNA in the cytoplasm due to using transient transfection for that experiment could have stimulated IIRs in a subpopulation of cells. Although INM redistribution of STING during IIRs has not been investigated, STING accumulates in perinuclear foci upon IIR activation with dsDNA but not dsRNA (Franz et al., 2018; Ishikawa et al., 2009). During this process, we observe a visible decrease in STING NE localization (Figure S2), implying that the nuclear pool may also contribute to these foci. As measurement of STING dynamics required using the STING-GFP cell line, we first compared the redistribution of the tagged and endogenous STING using two different antibodies and fixations while stimulating IIRs with either plasmid DNA or the dsRNA mimic poly(I:C), finding a similar redistribution pattern for the dsDNA and a similar lack of redistribution for poly(I:C) (Figures S2A–S2D).

**Figure 2:**
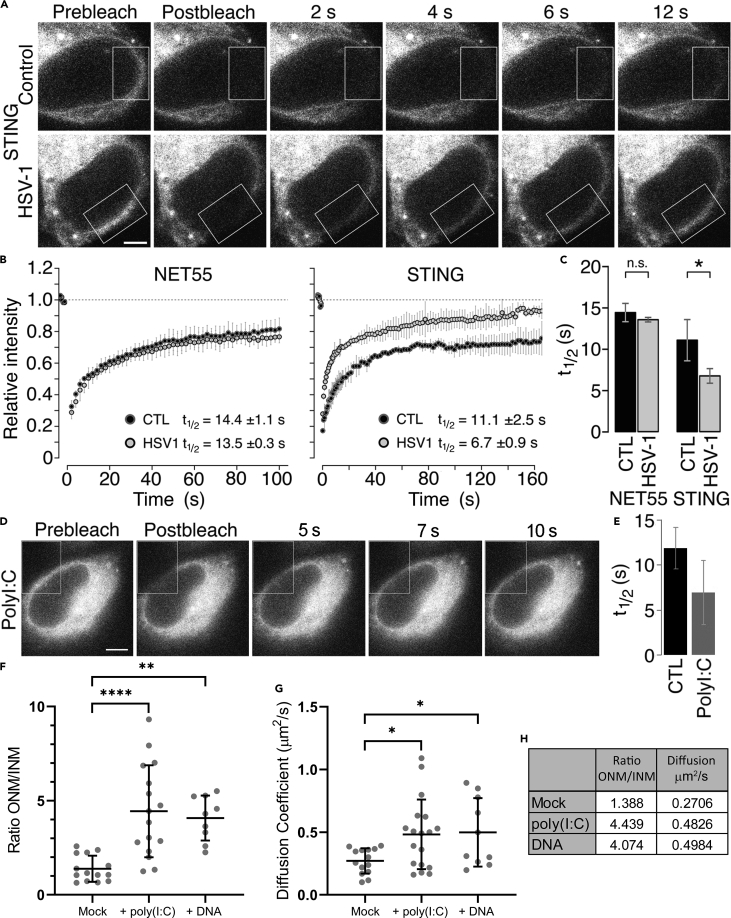
STING nuclear membrane mobility increases upon IIR activation (A) FRAP of STING-GFP in control (mock infected) and HSV-1-infected cells (2 hpi), photobleaching an area within the white outlined box. Scale bar, 5 μm. (B) Fluorescence recovery curves from three replicate experiments as in (A). Another NE protein, NET55, is shown as a control that does not change its dynamics with HSV-1 infection. CTL, control; HSV1, HSV-1 infected. Error bars show ± standard deviation. (C) Bar plot comparing the average half recovery times (t_1/2_) between the control and HSV-1-infected cells (student’s t test, ∗p ≤ 0.05). Error bars show ± standard deviation. (D) FRAP of STING-GFP in cells 2 hr after poly(I:C)-treatment, photobleaching an area within the white outlined box. Scale bar = 5 μm. (E) Bar plot comparing the average half recovery times (t_1/2_) between the untreated control and the poly(I:C)-treated cells. Error bars show ± standard deviation. (F) smFRAP microscopy on control and poly(I:C)- or dsDNA-transfected cells expressing STING-GFP revealed a redistribution from the inner nuclear membrane to the outer nuclear membrane/ER compartment. The ratio of particles in the outer nuclear membrane (ONM) over the inner nuclear membrane (INM) is plotted. Mean value shown, error bars show ± standard deviation. Statistics used ordinary one-way ANOVA with Dunnett’s multiple comparisons test, ∗∗p ≤ 0.01, ∗∗∗∗p ≤ 0.0001. (G) Measurement of mobility in the form of the diffusion coefficient measured in the same smFRAP microscopy experiments as in (F) revealed also increased mobility induced by polyI:C or dsDNA. Mean value shown, error bars show ± standard deviation. Statistics used ordinary one-way ANOVA with Dunnett’s multiple comparisons test, ∗p ≤ 0.05. (H) Table of summary data from (F) and (G) displaying mean values.

We used fluorescence recovery after photobleaching (FRAP) to test if IIR activation promotes STING shuttling between the nucleus and cytoplasm. For a control that would similarly avoid unintentional IIR induction due to transfected plasmid DNA, we generated a matched NET55-GFP-expressing HT1080 cell line. Induction of IIRs by infection with herpes simplex virus type 1 (HSV-1) visibly increased the speed of fluorescence recovery (Figure 2A), reducing the t½ for STING in the NE by ∼⅓ from 11.1 to 6.7 s while the t½ of control NET55 was unaffected (Figures 2B and 2C). This most likely indicates increased STING shuttling upon IIR activation because NE FRAP principally measures translocation through the peripheral NPC channels (Zuleger et al., 2011). HSV-1-induced STING mobility did not yield perinuclear foci as occuring with dsDNA stimulation, consistent with reports that HSV-1 inhibits STING activation and can prevent its translocation to the Golgi (Christensen et al., 2016; Pan et al., 2018; Zhang et al., 2016). Suprisingly, poly(I:C) transfection also increased STING mobility in the NE from control t½ 13.3 s to + poly(I:C) 7.59 s (Figures 2D and 2E). This was unexpected because the STING perinuclear foci only occur in response to DNA stimuli and not to poly(I:C), suggesting different functional pathways are involved.

We next turned to a different super-resolution approach, single-molecule fluorescence recovery after photobleaching (smFRAP) microscopy, which enables the tracking of individual NETs as they diffuse along the INM and ONM of the NE (Mudumbi et al., 2016, 2020; Tingey et al., 2019). The nuclear pool of STING-GFP redistributed out of the INM upon stimulation of the cells with poly(I:C) or dsDNA (Figure 2F). In unstimulated cells, a similar number of STING-GFP molecules were in the INM as the ONM, matching our immunogold EM data, while the ONM-to-INM ratio more than doubled in the poly(I:C)- and dsDNA-stimulated cells (Figures 2F and 2H). Measuring the diffusion coefficient of this mobile STING revealed a near doubling of the speed of particles from 0.27 to 0.48 μm^2^/s in the poly(I:C)-treated cells and to 0.49 μm^2^/s in dsDNA-treated cells (Figures 2G and 2H). Thus, induction of IIRs using plasmid dsDNA, infection with a dsDNA virus, or a dsRNA mimic all increase the mobility of STING in the NE even though the outcomes of the treatments differ with only the plasmid dsDNA treatment resulting in the accumulation of perinuclear foci. Moreover, although we did not test the dsDNA virus infection for single-molecule movements owing to health and safety restrictions, both plasmid dsDNA and the dsRNA mimic treatments resulted in a movement of STING molecules from the INM to the ONM, from whence this nuclear evacuated pool of STING could subsequently move throughout the cytoplasmic membrane systems.

### Many STING NE partners are nucleotide-binding proteins

**Figure 3:**
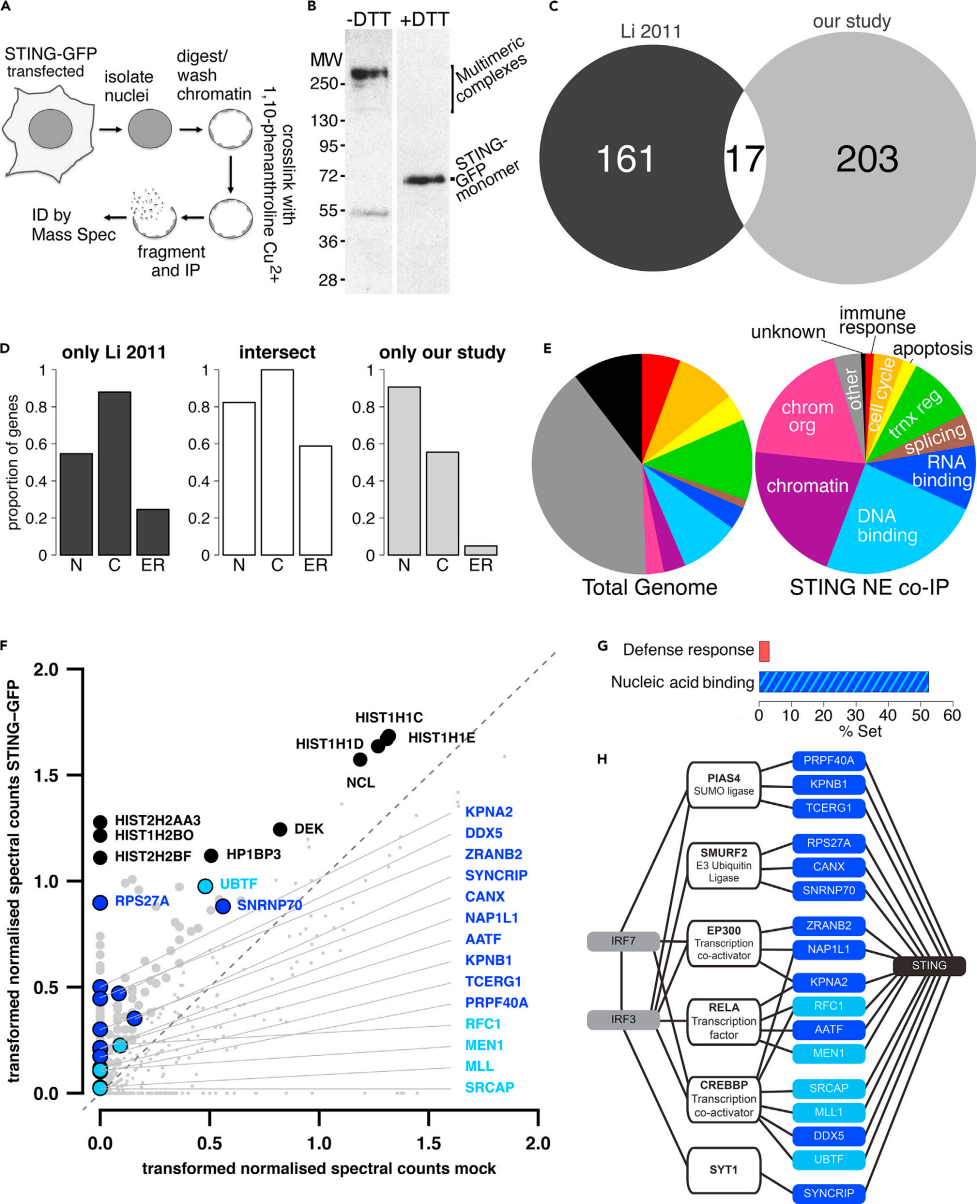
Many proteins identified by STING NE co-IP have nucleotide-binding functions (A) Schematic of reversible-cross-linking approach. NEs were isolated from HEK293T cells expressing STING-GFP or mock transfected cells. The NEs were cross-linked with *ortho*-phenanthroline copper, fragmented by sonication and STING-GFP cross-linked proteins recovered by immunoprecipitation with GFP antibodies. The cross-linking was reversed to release these other proteins and their identity determined by mass spectrometry. (B) Cross-linking of NEs with *ortho*-phenanthroline copper chases most STING-GFP to multimeric species >200 kDa, while a smaller portion appears at 55 kDa presumably owing to intramolecular cross-links. DTT-induced reversal of cross-linking restores all STING-GFP to its expected molecular weight at ∼69 kDa. (C) STING partner proteins identified in a previous co-IP study that should have preferentially identified cytoplasmic partners (Li et al., 2011) and here, where NE partners were specifically sought, were compared using a Venn diagram, finding that <8% of proteins found in this study were also found in the other study. (D) Gene ontology (GO) localization classification for STING putative NE partners in the two studies. The proportion of all genes in each set with GO term classifications for the nucleus, cytoplasm, and ER was plotted. (E) Gene ontology (GO) biological process classification for STING putative NE partners identified by mass spectrometry of cross-linking NE co-IP material. The representation of the GO-terms by the number of genes in the total human genome is shown on the left, while on the right are the terms as represented in the STING co-IP material with weighting based on the number of spectra recovered from each protein. (F) Putative STING NE partners plotted on log scale by normalized spectral abundance and enrichment in STING-GFP samples versus control samples. Nearly all of the most abundant partners were histone H1 variants followed by DNA-/RNA-binding proteins and bromodomain proteins. The position of the proteins indicated by the analysis in panel (H) is highlighted in blue. (G) Bar graph showing the representation within the set of putative STING NE partners of all GO terms associated with host defense responses or nucleic acid binding. (H) Known interacting proteins for the putative STING NE partners identified from the reversibly cross-linked NEs were searched for using the HPRD interactome database. Seventeen of the putative STING NE partners (blue) had reported interactions with 6 proteins (white boxes) reported to bind IRF3/7 transcription factors (gray) central to IIR activation.

Having established that STING is present in the INM, we sought to investigate its role in the nucleus by identifying NE-specific partners that may have been missed in earlier studies because STING’s association with the intermediate filament lamin polymer renders this pool highly insoluble (Malik et al., 2010). To maintain interactions with potential NE partner proteins when subsequently disrupting STING’s strong association with the INM and nuclear lamina, NEs were first isolated from HEK293T cells transiently expressing STING-GFP and then treated with a reversible cross-linker. Cross-linking chased STING-GFP into complexes between 130 and 300 kDa that could be reverted to the expected 70 kDa upon reversal of the cross-linking with DTT (Figures 3A and 3B). Cross-linked NEs were fragmented by sonication, immunoprecipitated (IP'd), cross-links reversed, and putative partners identified by tandem mass spectrometry (Table S1). We selected a high-confidence set of putative partners based on having at least 2 spectra and the normalized spectral score being at least 2-fold enriched over a control mock-transfected sample (orange highlighted in Table S1). Notably, there was <8% overlap between the proteins we identified and those identified in an earlier study of STING partners (Li et al., 2011) (Figure 3C), further attesting to the failure of standard co-IP approaches to recover the NE pool of proteins that associate with the lamina. Moreover, plotting the proportion of genes in each data set with gene ontology (GO) cellular component terms revealed the previous STING partner data set to be enriched in proteins with known cytoplasmic and ER localizations while our STING partner data set was enriched in proteins with known nuclear localization and the small intersect population had >80% of proteins with known localization to both the nucleus and the cytoplasm (Figure 3D).

The proteins that co-IP'd in the STING-cross-linked NEs were weighted for likely abundance based on spectral counts and plotted based on GO biological process terms. This revealed enrichment in proteins with GO terms for chromatin/chromosome organization and RNA/DNA binding compared with their representation among all proteins encoded by the genome (Figure 3E). Plotting the normalized spectral abundance in the STING-GFP sample compared with mock transfected cells (Figure 3F) revealed the most abundant of the enriched co-IP proteins to be histone H1 variants followed by a mixture of known NE proteins (e.g., Lamin A, LAP2), nucleotide-binding proteins (e.g., snRNP70, UBTF, RPS27a), and bromodomain proteins (e.g., Brd2, Brd3, Rbmx) that could mediate the reported STING function in chromatin compaction (Malik et al., 2014) and other epigenetic changes associated with IIRs (Mehta and Jeffrey, 2015) (Table 1). Many proteins in all these categories bind DNA/RNA and nearly half of all STING partners identified are listed as nucleotide-binding proteins (Figure 3G). Strikingly, although some known STING interactors were identified in the NE-STING proteome, e.g., DDX41 (Zhang et al., 2011) and CCDC47 (Li et al., 2011), many well-known interactors such as TBK1 and MAVS were not found, suggesting that the NE-STING proteome differs significantly from that of STING localized in the ER.

**Table 1:** Functional groups of STING highest abundance NE interactors based on raw spectral counts

Epigenetic	s	NET/NE	s	Histone	s	RNA	s	Other	s
HP1B3	77	LMNA	48	HIST1H1C	104	PSIP1	50	NCL	287
BRD2	64	TMPOβ	28	HIST1H1E	103	SNRNP70	35	UBF1	78
KIAA0020	50	TMPOα	18	HIST1H1D	97	RBM28	33	DEK	73
BRD3	42	CKAP4	15	HIST2H2AA3	26	HNRNPG	31	MFAP1	47
BAZ2A	28	KPNA2	13	HIST1H2BO	22	EBNA1BP2	30	CD11B	46
MECP2	19			HIST2H2BF	17	RRP1B	24	RL1D1	41
HELLS	16					HNRNPR	23	CCDC86	38
RSF1	12					HNRNPL	18	NOP2	23
						PAF1	18	VRK1	21
						HNRNPK	17	ILF3	16
						RRMJ3	15	KIF22	16
						RBMXL1	15	HDGR2	15
						DDX5	14	NOP58	14
						SRSF2	13	GTF2I	14
						RPS27A	13	UHRF1	13
								HSPA1A	13

NOTE: restricted to those with >3x more spectra (s) in STING sample than in mock sample.

Downstream of STING ER/Golgi functions, IRF3/7 transcription factors induce IFN and other IIR genes in the nucleus. Therefore, we wondered if some of these STING NE co-IP partners have known interactions with IRF3/7 and may modulate immune-signaling cascades. Accordingly, we searched the HPRD interactome database (Peri et al., 2003) using Cytoscape (Lopes et al., 2010), finding that IRF3/7 had no known direct interactions with any of the putative STING partners. However, six known direct IRF3/7-binding partners interact directly with 17 of the proteins identified in the STING-NE co-IP (Figure 3H). Of these, 12 are RNA-binding proteins (dark blue). The rest, as well as some of the RNA-binding proteins, have also been reported to bind DNA. Although these proteins have not been previously shown to affect IRF3/7 transcriptional responses in IIRs, several interact with viral proteins and affect viral replication and so may contribute to host cell IIR. For example, DDX5 is bound by the N(pro) protease of pestivirus (Jefferson et al., 2014a, 2014b) and may inhibit hepatitis C virus replication (Upadya et al., 2014) and vesicular stomatitis virus triggered IFNβ induction (Zheng et al., 2017), although it appears to be a positive regulator of HIV-1 (Zhou et al., 2013) and Japanese encephalitis virus (JEV) (Li et al., 2013a), among others (Cheng et al., 2018). Meanwhile, the hepatitis B virus HBx protein alters the intracellular distribution of RPS27a (Fatima et al., 2012), AATF is specifically targeted by an HIV-encoded miRNA (Kaul et al., 2009), and SYNCRIP is involved in hepatitis C virus replication (Liu et al., 2009) and mouse hepatitis virus RNA synthesis (Choi et al., 2004). Therefore, we postulated that proteins identified in the NE STING co-IP experiment could contribute to IIR signaling and the potential links to IRF3/7 transcription factors suggested a signaling network through which STING might influence IIRs from the INM.

### STING NE co-IP partners contribute to IIRs

**Figure 4:**
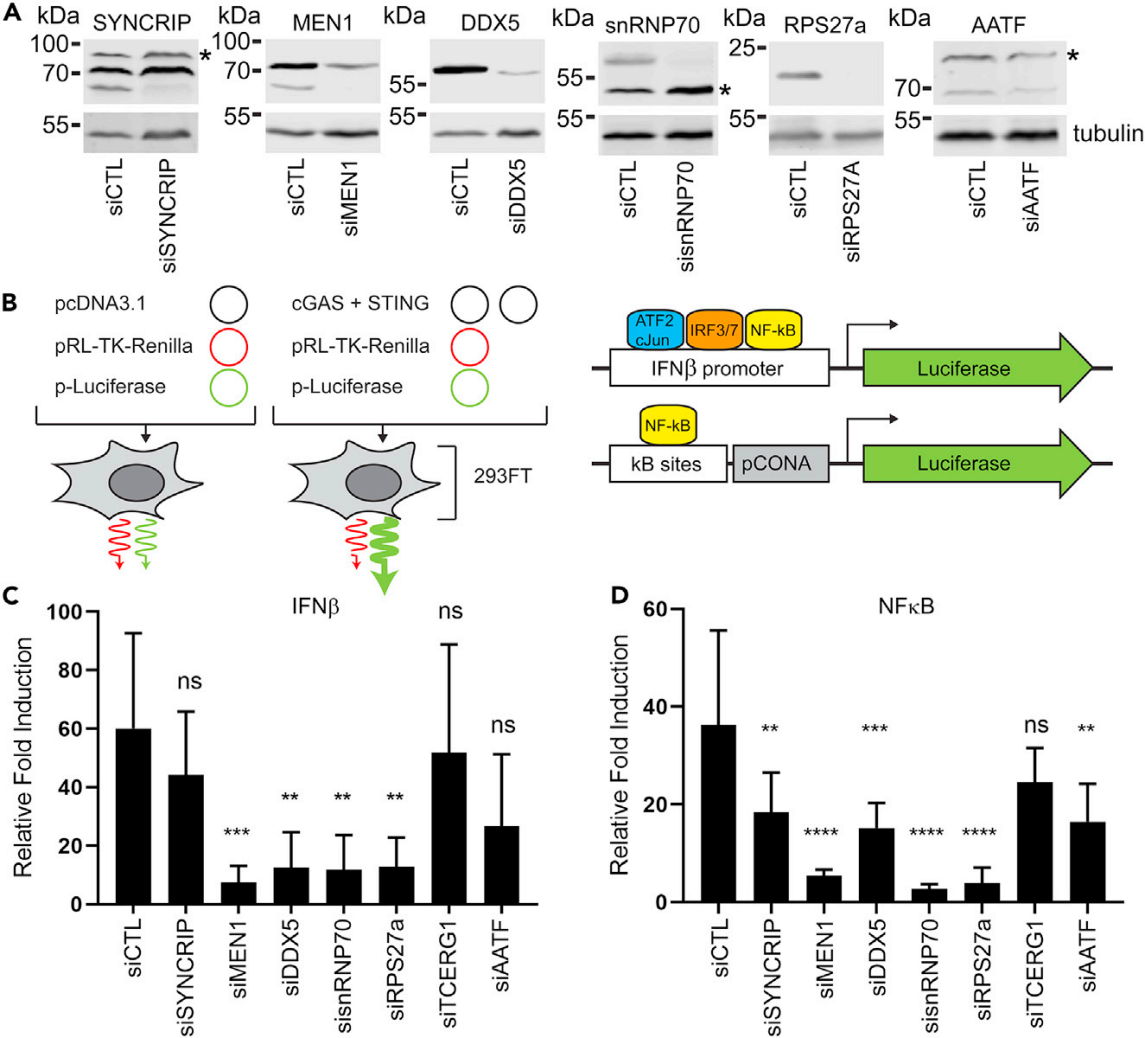
STING putative NE partners contribute to IIR activation (A) Confirmation of siRNA knockdowns for testing effects of partners in IIR activation assays. Representative Western blots for partners with antibodies that detected proteins of expected molecular weight are shown. ∗ indicates nonspecific bands recognized by antibody. In the case of SYNCRIP, the highest molecular weight band likely represents the homologous hnRNP R protein which shares a large degree of sequence identity with SYNCRIP and is reported to be recognized by anti-SYNCRIP antibodies. (B) Schematic of dual luciferase assay used to measure activity of IIR reporter genes. Plasmids expressing Renilla Luciferase variant under a thymidine kinase promoter and Firefly Luciferase under a promoter activated by NF-κB binding or the IFNβ promoter are transfected with or without cGAS and STING into 293FT cells. These cells do not express cGAS and have low levels of endogenous STING, so the transfection induces IIRs in a controlled manner. Comparing the Renilla and Firefly Luciferase levels further controls for differences in the transfection efficiency and cell number. (C) IF-Nβ promoter reporter reveals a significant reduction in the IIR activation when 4 of the 7 STING putative NE partners were knocked down. Six replicates were performed and analyzed with ordinary one-way ANOVA and Dunnett’s multiple comparisons test, ∗∗p ≤ 0.01, ∗∗∗p ≤ 0.001. Mean values shown with ± standard deviation. (D) NF-κB-activated reporter reveals a significant reduction in the IIR activation when 6 of the 7 STING putative NE partners were knocked down. Six replicates were performed and analyzed with ordinary one-way ANOVA and Dunnett’s multiple comparisons test, ∗∗p ≤ 0.01, ∗∗∗p ≤ 0.001, ∗∗∗∗p ≤ 0.0001. Mean values shown with ± standard deviation.

To test whether putative partner proteins identified in the NE STING co-IP experiment are involved in dsDNA-triggered IIRs, we used a dual-luciferase reporter system in combination with siRNA-mediated knockdown of 7 partner proteins with links to IRF3/7 (Figure 4A) to test for effects on expression of an IFNβ promoter-driven reporter or a reporter activated by NF-κB binding. The NFκB- and IFNβ-luciferase reporters are activated upon cotransfection of STING and cyclic GMP-AMP synthase (cGAS) (Figure 4B). cGAS produces a second messenger (cGAMP) that is bound by STING during IIRs (Sun et al., 2013), and HEK293FT cells were used because they do not express cGAS (Figure S1E) so that the only source was the transfected plasmid. The cells were also cotransfected with a Renilla luciferase reporter under a thymidine kinase promoter to allow for normalization of transfection efficiency and cell number. Using this assay, siRNA knockdown of MEN1, DDX5, snRNP70, and RPS27a all caused a statistically significant drop in IFNβ-promoter-driven luciferase expression (Figure 4C), while knockdown of SYNCRIP, MEN1, DDX5, snRNP70, RPS27a, and AATF all exhibited a statistically significant drop in NF-κB-activated luciferase expression (Figure 4D). This suggests that these putative STING partner proteins can themselves contribute to IIRs. It is interesting that SYNCRIP and AATF were more restricted in only being able to affect luciferase expression from the NF-κB-driven reporter.

**Figure 5:**
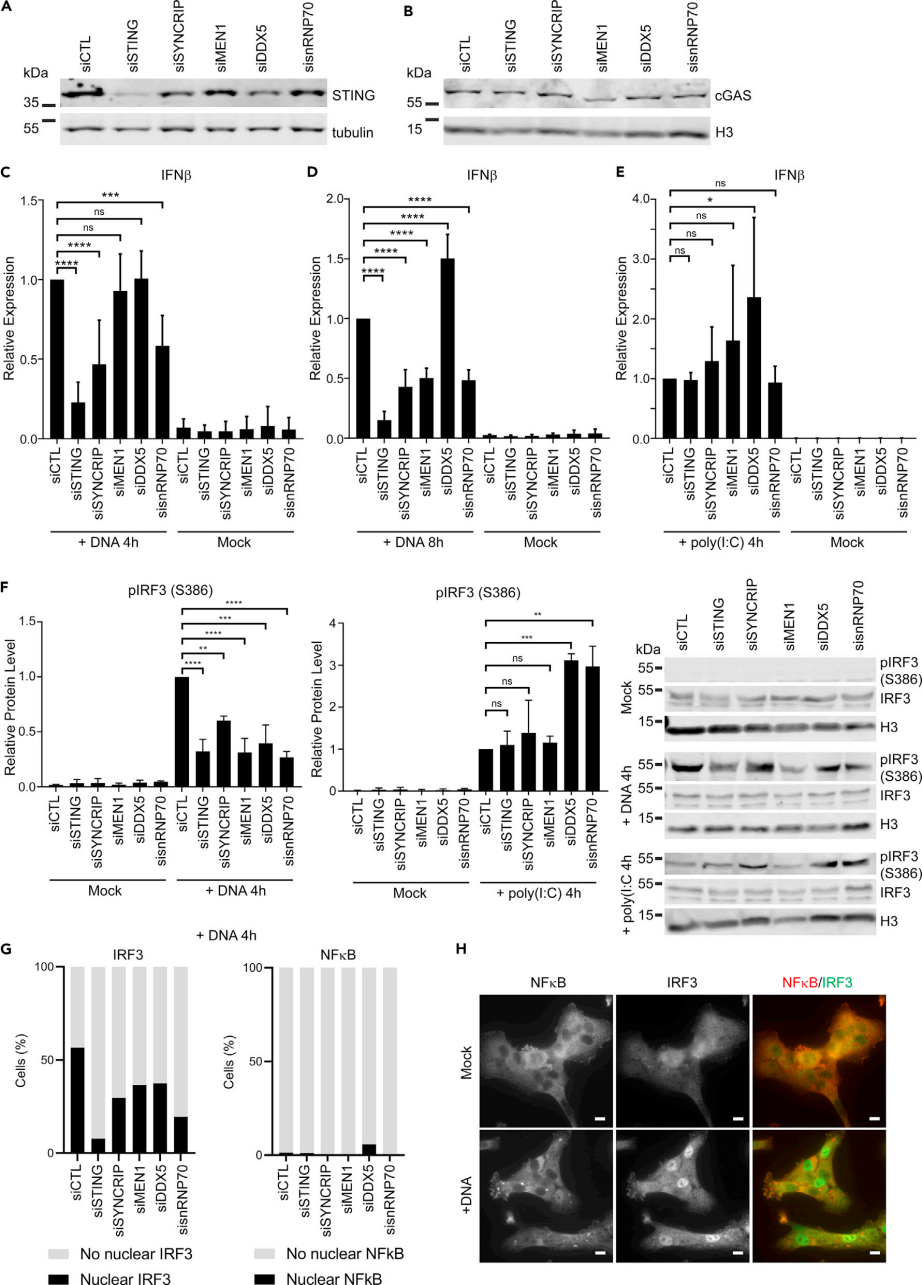
STING putative NE partners have stronger effects on dsDNA-stimulated than dsRNA-stimulated IIRs Contributions of STING putative NE partners to IIRs were further confirmed by measuring effects on IIRs induced by treatment with dsDNA or poly(I:C), dsRNA mimic. These assays were performed in HT1080 cells that express endogenous STING and cGAS. (A and B) Western blotting confirms minimal effects of siRNAs on STING and (B) cGAS expression, except for siDDX5 which caused a modest reduction in STING protein levels. Representative blots of three independent experiments. (C) Quantification of IF-Nβ transcripts by qPCR reveals strong effects of STING putative partners, SYNCRIP and SNRNP70 4 hr after transfection of dsDNA (n = 3). Mean values shown with ± standard deviation (ordinary one-way ANOVA with Dunnett’s multiple comparisons test ∗ ≤0.05, p ∗∗p ≤ 0.01, ∗∗∗p ≤ 0.001, p ∗∗∗∗ ≤0.0001). (D) Effects on IF-Nβ transcripts are greater 8 hr after transfection of dsDNA. SYNCRIP, MEN1, and SNRNP70 siRNAs all reduced IFNβ transcripts, while DDX5 siRNA treatment caused a significant increase in IF-Nβ transcripts relative to siRNA-control-treated samples (n = 3). Mean values shown with ± standard deviation (ordinary one-way ANOVA with Dunnett’s multiple comparisons test ∗ ≤0.05, p ∗∗p ≤ 0.01, ∗∗∗p ≤ 0.001, p ∗∗∗∗ ≤0.0001). (E) In contrast, no effect was observed in response to poly(I:C), except for DDX5 knockdown which caused a significant increase in IF-Nβ levels (n = 3). Mean values shown with ± standard deviation (ordinary one-way ANOVA with Dunnett’s multiple comparisons test ∗ ≤0.05, p ∗∗p ≤ 0.01, ∗∗∗p ≤ 0.001, p ∗∗∗∗ ≤0.0001). (F) Effect of partner protein knockdown on IRF3 phosphorylation (pIRF3) after immune stimulation with dsDNA or poly(I:C). Western blotting representative of three independent experiments. Mean values shown with ± standard deviation (ordinary one-way ANOVA with Dunnett’s multiple comparisons test ∗ ≤0.05, p ∗∗p ≤ 0.01, ∗∗∗p ≤ 0.001, p ∗∗∗∗ ≤0.0001). (G) Quantification of the number of cells with accumulation of IRF3 or NF-κB transcription factors in the nucleus after treatment with siRNAs against STING and putative NE partners and immune stimulation with dsDNA (4 hr after transfection) (n ≥ 100 cells). (H) Representative images for IRF3 and NF-κB immunofluorescence used to quantify percentage of cells with nuclear accumulation of IRF3 and NF-κB in (G).

To further confirm the role of these STING NE co-IP partners in IIRs, independent of the luciferase assay system, we measured transcripts of IFNβ with the various knockdowns in HT1080 cells (Figure S3A) ± initiation of IIRs with plasmid DNA or poly(I:C). HT1080 cells have a functional cGAS-STING pathway as shown by the redistribution of STING into perinuclear foci upon dsDNA-triggered immune stimulation and the accumulation of IRF3 in the nucleus (Figures S2A and S2B). These cells also respond to dsRNA-triggered immune stimulation as shown by the accumulation of IRF3 in the nucleus following poly(I:C) transfection, indicating that RIG-I/MDA-5/MAVS signaling pathways are functional (Figure S2A). siRNA knockdown of STING partners SYNCRIP, MEN1, and SNRNP70 did not affect STING or cGAS protein levels as determined by Western blot (Figures 5A and 5B). However, knockdown of DDX5 caused a modest reduction in STING protein levels, suggesting that either DDX5 is required for STING stability, or an off-target effect of the siRNA used. As expected, STING knockdown strongly reduced the amount of IFNβ induction upon plasmid DNA but not poly(I:C) stimulation of IIRs (Figures 5C–5E). SYNCRIP, MEN1, and snRNP70 knockdown all reduced IFNβ induction by more than 50% (Figures 5C and 5D), an effect that was more marked at 8 h after DNA transfection. Surprisingly, DDX5 knockdown significantly enhanced IFNβ induction with both DNA and poly(I:C) immune stimulation. This effect is especially interesting given that DDX5 siRNA treatment caused a reduction in STING protein levels and suggests that DDX5 functions as a negative regulator of both DNA- and RNA-triggered IIRs. Surprisingly, despite that several of these STING NE coIP partners are RNA-binding proteins, when IIR was induced with poly(I:C), the other proteins tested did not have a significant effect on IFNβ induction, suggesting that they specifically modulate IFNβ induction during DNA-triggered immune responses (Figure 5E).

We wondered if the STING redistribution observed in Figure 2 might be part of a mechanism through which these STING partners mediate IIRs. Therefore, we tested for altered STING redistribution between the nucleus and ER upon IIR activation when different partners were knocked down. Isolated nuclei and microsomes (representing ER) were prepared and tested for relative levels STING in the two compartments by Western blot (Figure S4). In mock stimulated cells, little difference was observed for STING localization between NE and microsome fractions with different partner knockdowns compared with the control with ∼70–75% of STING present in the NE fraction. The exception was with MEN1 knockdown, which resulted in ∼50% of STING present in each fraction. In dsDNA-stimulated cells, STING localization between NE and microsome fractions was largely unchanged, although there was a slight reduction in STING present in the NE fraction in the control and SYNCRIP knockdown conditions, ∼60% in the NE fraction, potentially reflecting the redistribution of STING from the INM observed by smFRAP (Figure 2F). In poly(I:C)-stimulated cells, STING present in the NE was reduced to ∼50% for control and SYNCRIP knockdown cells, again consistent with smFRAP data showing a redistribution of STING from the INM in poly(I:C)-stimulated cells (Figure 2F). Interestingly, in MEN1 knockdown cells stimulated with poly(I:C), STING present in the NE fraction was increased to ∼75%, while in DDX5 knockdown cells, STING present in the NE fraction remained ∼70% across all tested conditions. However, how these slight changes on STING localization with partner knockdown could impact the mechanism of their contributions to IIRs is unclear.

Given the potential links between NE STING partners and IRF3/7 and the effects on IFNβ induction, we decided to look at whether their knockdown affected IRF3 activation as measured by IRF3 phosphorylation. IRF3 phosphorylation upon treatment with plasmid DNA was reduced when STING or its NE co-IP partners were knocked down compared with cells treated with control siRNA (Figure 5F). In contrast, with poly(I:C) treatment, there were no obvious effects on IRF3 phosphorylation in cells knocked down for STING and MEN1; however, it was significantly enhanced in cells knocked down for DDX5 and snRNP70, while also slightly increased in cells knocked down for SYNCRIP although not at a statistically significant level (Figure 5F). As another measure of IRF3 activation, cells treated with siRNAs against STING and partner proteins were assayed for accumulation of IRF3 in the nucleus by microscopy. In agreement with the reduction in phosphorylated IRF3 seen in cells treated with siRNAs against STING and partners after immune stimulation with dsDNA, the percentage of cells positive for accumulation of IRF3 in the nucleus was reduced in all conditions compared with cells treated with a control siRNA (Figures 5G and S3B). Nuclear accumulation of NF-κB (p65) was also tested, revealing that in response to dsDNA treatment p65 is only weakly activated (phosphorylated p65 accumulates in the nucleus) in HT1080 cells (Figures 5G and 5H). In contrast, treatment of knockdown cells with poly(I:C) led to a robust activation of IRF3 and NF-κB, as determined by the accumulation of NF-κB (p65) in the nucleus (Figure S3C). Collectively, these data suggest that SYNCRIP, MEN1, and SNRNP70 are positive regulators of dsDNA-stimulated IIRs while DDX5 is a negative regulator of dsDNA- and dsRNA-stimulated IIRs.

### STING NE co-IP partner SYNCRIP is antiviral against influenza A virus

**Figure 6:**
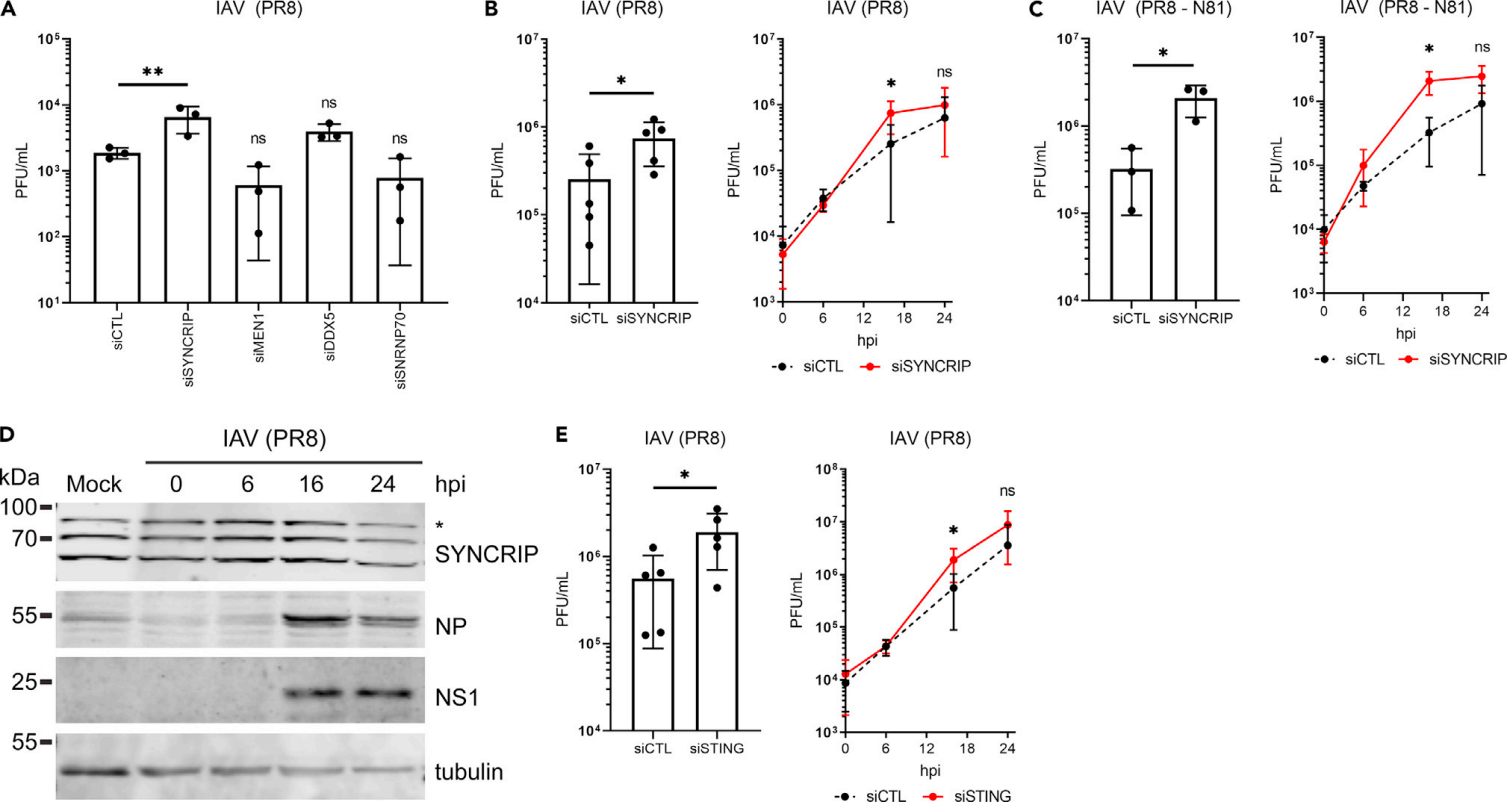
SYNCRIP antagonizes IAV infection (A) Determination of viral titers in cell culture medium collected from HT1080 cells knocked down for STING partners, 24 hr after infection (hpi) with IAV (PR8 strain) at a multiplicity of infection (MOI) of 0.01, by plaque assay (PFU = plaque-forming units) (n = 3). Mean values shown with ± standard deviation (ordinary one-way ANOVA with Dunnett’s multiple comparisons test ∗∗p ≤ 0.01). (B) Confirmation of SYNCRIP knockdown effect on IAV titers in cells infected with a higher multiplicity of infection (MOI = 3). The left panel shows significantly higher viral titers at 16 hpi, and the right panel shows time course of infection (n = 5). Mean values shown with ± standard deviation (student’s t test, ∗p ≤ 0.05). (C) Effect of SYNCRIP knockdown on viral titers is stronger on IAV mutant virus with truncated NS1 protein (NS1-N81) (MOI = 3). The left panel shows significantly higher viral titers at 16 hpi, and the right panel shows time course of infection (n = 3). Mean values shown with ± standard deviation (student’s t test, ∗p ≤ 0.05). (D) Western blotting of SYNCRIP (∗ indicates nonspecific band) and viral proteins, NP and NS1, during IAV infection shows no obvious effect on SYNCRIP protein levels. (E) Confirmation that STING knockdown is beneficial to IAV infection in HT1080 cells as determined by increased viral titers relative to siRNA-control-treated cells (MOI = 3). The left panel shows significantly higher viral titers at 16 hpi, and the right panel shows time course of infection (n = 3). Mean values shown with ± standard deviation (student’s t test, ∗p ≤ 0.05).

Although none of the STING co-IP partners tested were found to have negative effects on poly(I:C)-stimulated IFN expression (Figure 5E), we decided to test whether they might play a role in IIR against an RNA virus because STING may function in IIRs triggered by RNA virus infection in a manner independent of IFN induction (Ishikawa et al., 2009; Jin et al., 2008; Sun et al., 2009). After siRNA-mediated knockdown of STING NE partners, HT1080 cells were infected with the nuclear replicating RNA virus, influenza A virus (IAV). Knockdown of SYNCRIP resulted in significantly higher viral titers as determined by plaque assays, both at low and high multiplicity of infection (MOI) (Figures 6A and 6B). This effect was stronger for a mutant IAV, PR8 – N81 (Pereira et al., 2017), which expresses an NS1 protein with a deletion of the effector domain and so is less able to antagonize host IIRs (Figure 6C). To determine whether SYNCRIP expression is altered during viral infection, cell lysates were harvested at multiple time points during infection and blotted for SYNCRIP, revealing no obvious difference in SYNCRIP protein levels during infection (confirmed by the presence of viral proteins NP and NS1) compared with mock infected cells (Figure 6D). STING knockdown was previously shown to affect IAV replication (Holm et al., 2016; Moriyama et al., 2019), and we replicated this here in HT1080 cells. Knockdown of STING resulted in significantly higher viral titers than cells treated with a control siRNA (Figure 6E). Furthermore, we found that the phenotypic effects of SYNCRIP knockdown replicate those of STING knockdown.

## Discussion

In the best characterized "canonical" STING pathway, recognition of cytoplasmic dsDNA by cGAS triggers cGAMP production which associates with STING to promote its activation. Activated STING dimers translocate from the ER to the Golgi where they accumulate in perinuclear foci (Burdette et al., 2011; Ishikawa and Barber, 2008; Ishikawa et al., 2009; Saitoh et al., 2009; Sun et al., 2013; Wu et al., 2013). From here STING dimers oligomerize, inducing TANK-binding kinase 1 (TBK1) activation which *trans* phosphorylates itself and neighboring STING dimers (Liu et al., 2015; Tanaka and Chen, 2012; Zhong et al., 2008), leading to the recruitment and activation of IRF3 and eventually the induction of type-I IFNs and proinflammatory cytokines. However, STING translocation to or from the nucleus in IIRs was previously unknown. Our finding that the INM STING pool translocates out of the nucleus upon IIR activation is particularly intriguing in light of recent reports that most cGAS is in the nucleus (Ablasser and Chen, 2019; Gentili et al., 2019; Ng et al., 2018; Zierhut et al., 2019), raising the possibility that INM STING could be activated before ER STING after detection of nuclear-localized viral dsDNA. Indeed, our finding that STING mobility in the NE increases during infection with the dsDNA nuclear-replicating virus HSV-1 would support this notion. This finding also has implications for a recent report of cGAS-independent activation of STING after detection of DNA damage, in which the authors propose a noncanonical signaling complex composed of STING, TRAF6, IFI16, and p53 that forms in response to DNA damage sensed by PARP1 and ATM and initiates an NF-κB-dominated transcriptional response (Dunphy et al., 2018). It is possible that such a signaling complex forms in the nucleus given that the authors of this study reported no redistribution of ER-resident STING to perinuclear foci after the induction of DNA damage by etoposide. It is interesting in this regard that one of the more abundant STING NE coIP hits was the DNA damage response protein PARP1 (see Table S1). Although this was not included as a top hit because of our requirement that there be twice as many spectra in the STING sample as in the mock (PARP1 was 106 to 62), this and other proteins identified in the proteomics further support a role of nuclear STING functioning to sense nuclear DNA damage to induce immune responses in cancer.

Furthermore, our finding that poly(I:C) increases STING mobility while promoting its redistribution away from the INM suggests a possible mechanism for STING protection against RNA viruses. This function seems to use a noncanonical pathway because STING does not redistribute from the ER to Golgi perinuclear foci with poly(I:C) treatment. Because STING is reported to not directly bind RNA or poly(I:C) (Abe et al., 2013), these partners might mediate interactions with foreign RNA. It was recently reported that STING restricts the replication of RNA viruses through a proposed mechanism dependent on the cytosolic dsRNA sensor RIG-I and due to a general inhibition of translation independent of PKR (Protein kinase R) and translocon functions (Franz et al., 2018). Several of the STING NE partners we identified here could potentially mediate STING effects on translation (e.g., RPS27a, SYNCRIP, snRNP70); however, this does not preclude the possibility that these partners provide specific recognition of different RNA viruses and thus serve to enhance the variety of IIR nucleotide sensors/adaptors. Interestingly, despite having no effect on poly(I:C)-mediated interferon expression, we find SYNCRIP to play a role in antagonizing IAV. Whether this is through a general translation effect, or indirectly through the cGAS-STING pathway stimulated by mitochondrial stress and DNA leakage (as reported for other RNA viruses over the years (Aguirre and Fernandez-Sesma, 2017; Machida et al., 2006)), remains to be determined.

That the INM STING pool can mobilize to translocate to the ONM through the peripheral channels of the NPC may reflect a backup mechanism to signal IIRs using the peripheral NPC channels when viruses inhibit the central channel transport. Viruses often target the central channel of the NPCs to either block transport or usurp it so that virus transcripts are preferentially transported over host-directed transport (Gustin, 2003; Petersen et al., 2001; Tessier et al., 2019; Yarbrough et al., 2014), but the peripheral channels are normally used for membrane protein transport (Mudumbi et al., 2016, 2020; Soullam and Worman, 1995; Zuleger et al., 2011) so that STING as a multispanning transmembrane protein could bypass this block to signal IIRs. This does not preclude the well-established STING signaling cascades from the ER/Golgi compartment normally using the central channel of the NPC—indeed IRF3 is known to translocate through the NPC central channel (Lin et al., 1998; Zhu et al., 2015), but our findings of increased STING mobility and nucleo-cytoplasmic shuttling during IIRs through the peripheral NPC channels suggest that STING may provide a backup system for activating IIRs when the central channel transport is disrupted. Moreover, the 17 STING NE co-IP partners identified here that interact with six IRF3/7 partners could potentially contribute to such IIR activation, enhancing STING functions through a multiply redundant backup system.

The increasing complexity of STING interactions and its multiple pathways for activating IIRs make confirmation of STING's involvement in the IIR contributions of these STING partners difficult. Nonetheless, they clearly can contribute to IIRs from the measures shown here, and several reports in the literature support this when reevalutated in light of our results. MEN1 binds and represses the activity of the AP1 transcription factor JunD (Agarwal et al., 1999), and the related cJun is an IIR activator (Wathelet et al., 1998), possibly explaining its functioning in IIRs. MEN1 was also recently found to affect promoter fidelity at the interferon-gamma inducible IRF1 gene (Auriemma et al., 2012). Interestingly, this function of MEN1 involves its functioning in a complex with the major histone K4 methyltransferase, MLL1, which was also identified as an STING NE co-IP partner. STING interaction with this methyltransferase complex could also contribute to the other reported nuclear function for STING in chromatin compaction (Malik et al., 2014). In addition, several bromodomain proteins identified as putative STING partners here (e.g., BRD2, BRD3) could also explain this chromatin compaction function or, more excitingly, chromatin remodeling reported to occur in IIRs (Mehta and Jeffrey, 2015). Furthermore, MEN1 is a tumor suppressor (Fang et al., 2013) and as such could contribute to reported STING roles in DNA damage sensing in cancer (Ablasser and Chen, 2019; Gentili et al., 2019; Mackenzie et al., 2017; Ng et al., 2018; Qiu et al., 2020; Zierhut et al., 2019). A recent study showed that MEN1 depletion results in misregulation of the p53 pathway leading to increased levels of chromosomal instability and accumulation of DNA damage (Qiu et al., 2020).

The STING-NE co-IP partners that bind RNA also have several previously reported functions that would be consistent with their ability to support IIRs indicated here. For example, the DDX5 targeting by the N(pro) protease of pestivirus presumably counters host antiviral defenses (Jefferson et al., 2014a, 2014b). At the same time, DDX5 can be a negative regulator of IFN responses. Our finding that DDX5 knockdown increases type-I IFN expression after DNA or poly(I:C) transfection and IRF3 phosphorylation after poly(I:C) transfection is consistent with a recent report that DDX5 suppressed IFN responses triggered by VSV infection (Zan et al., 2020). This study also showed that DDX5 knockdown increased IRF3 phosphorylation, but without testing whether DDX5 knockdown influences IRF3 phosphorylation triggered by a DNA ligand as we do. Our findings here that DDX5 knockdown reduces dsDNA-induced IRF3 activation while elevating IFNβ expression might appear contradictory without the context of these other studies suggesting it can be both stimulatory and inhibitory to IIR induction. Regardless, these results strongly suggest that DDX5 may contribute a regulatory function to IIRs. While this study has focused on characterizing potential IIR activity of the specific binding partners highlighted for having upstream effects on IRF3/7 transcription factors, it is notable that many more of the top STING NE co-IP partners bind RNA and some have previously been shown to mediate IIRs. One of these is DDX23 that is a dsRNA sensor recently reported to pair with TRIF or MAVS to mediate IIRs (Ruan et al., 2019).

Other links to viral infection for these newly identified STING partners include the hepatitis B virus HBx protein that alters the intracellular distribution of RPS27a (Fatima et al., 2012). Additionally, AATF is specifically targeted by HIV to impair cellular responses to infection (Kaul et al., 2009). SYNCRIP/hnRNP Q interestingly facilitates hepatitis virus replication (Liu et al., 2009), suggesting an alternate pathway where STING sequestration of this factor might provide another avenue toward host protection from the virus. SYNCRIP was separately reported to interact with the IAV NS1 protein (Rahim et al., 2018), a major antagonist of the host cell immune response, suggesting the virus may target SYNCRIP owing to its positive immune functions. These many RNA-binding partners could provide a highly redundant backup system so that knockout of any single one would only moderately impact IIRs, consistent with the moderate but significant reduction in IIR signaling observed for SYNCRIP knockdown. Our data are consistent with the notion that STING plays a wider role in signaling than its initial description as an IIR adaptor in cytosolic DNA sensing, initiating different responses based on diverse inputs from DNA damage (Dunphy et al., 2018) to RNA virus infection (Franz et al., 2018). The wide range of STING nuclear partners combined with its ability to translocate out of the nucleus with treatments that activate IIR potentially provides a valuable redundancy and novel mechanism for STING functions that could better elucidate how it protects cells against both RNA and DNA viruses.

### Limitations of the study

Identifying partners of proteins in the INM is inherently tricky. This is because most such proteins bind to both chromatin and the intermediate filament lamin polymer while also being embedded in the membrane where they could also have luminal partners. Chromatin is inherently insoluble as is the lamin polymer that only becomes soluble in 7 M urea, and the solubilization conditions required to extract these proteins from the membrane and break INM proteins away from these structures are likely to break other partner protein interactions. While our reversible cross-linking approach is the best way we can find to get around these problems, it also has the limitation that there is a slightly higher possibility that partners identified could be indirect as part of larger complexes.

## Figures and Tables

**Table:** 

REAGENT or RESOURCE	SOURCE	IDENTIFIER
**Antibodies**		

Rabbit polyclonal anti-Lamin A	ECS lab	3262
Sheep polyclonal anti-STING	R&D Systems	Cat# AF6516; RRID:AB_10718401
Rabbit polyclonal anti-IRF3, FL-425	Santa Cruz Biotechnology	Cat# sc-9082; RRID:AB_2264929
Rabbit monoclonal anti-phospho-IRF3 (S386)	Abcam	Cat# ab76493; RRID:AB_1523836
Rabbit polyclonal anti-cGAS, D1D3G	Atlas Antibodies	Cat# HPA031700; RRID:AB_10601693
Mouse monoclonal anti-cGAS	Cell Signaling Technology	Cat# 15102; RRID:AB_2732795
Mouse monoclonal anti-histone H3	Abcam	Cat# ab10799; RRID:AB_470239
Mouse monoclonal anti-gamma tubulin, GTU-88	Merck	Cat# T6557; RRID:AB_477584
Sheep polyclonal anti-alpha tubulin	Cytoskeleton Inc.	Cat# ATN02; RRID:AB_10708807
Mouse monoclonal anti-SYNCRIP, 7A11.2	Millipore	Cat# MAB11004; RRID:AB_10806916
Rabbit polyclonal anti-MEN1	Abcam	Cat# ab2605; RRID:AB_303203
Goat polyclonal anti-DDX5	Abcam	Cat# ab10261; RRID:AB_297000
Rabbit polyclonal anti-snRNP70	Abcam	Cat# ab51266; RRID:AB_882630
Rabbit polyclonal anti-RPS27a	Abcam	Cat# ab172293
Rabbit polyclonal anti-AATF	Abcam	Cat# ab39631; RRID:AB_722512
Rabbit polyclonal anti-NS1	generated by PD	NS1-RBD
Rabbit polyclonal anti-NP	generated by PD	A2915
Rabbit polyclonal anti-Calnexin	Stressgen	Cat# SPA-860; RRID:AB_11178981
Rabbit monoclonal anti-STING, D2P2F	Cell Signaling Technology	Cat# 13647; RRID:AB_2732796
Mouse monoclonal anti-Nup153, QE5	Abcam	Cat# ab24700; RRID:AB_2154467
Mouse monoclonal anti-NFκB, L8F6	Cell Signaling Technology	Cat# 6956S; RRID:AB_10828935
Mouse monoclonal anti-Nup153, QE5	Covance	Cat# MMS-102P; RRID:AB_10642030
Rabbit polyclonal anti-Nup358	gift from Frauke Melchior	N/A
Rabbit polyclonal anti-GFP	Abcam	Cat# ab6556; RRID:AB_305564
Rabbit polyclonal anti-GFP	Life Technologies	Cat# A11122; RRID:AB_221569
Donkey anti-mouse Alexa Fluor 488	Thermo Fisher	Cat# A21202; RRID:AB_141607
Donkey anti-mouse Alexa Fluor 568	Thermo Fisher	Cat# A10037; RRID:AB_2534013
Donkey anti-mouse Alexa Fluor 594	Thermo Fisher	Cat# A21203; RRID:AB_141633
Donkey anti-mouse Alexa Fluor 647	Thermo Fisher	Cat# A31571; RRID:AB_162542
Donkey anti-rabbit Alexa Fluor 488	Thermo Fisher	Cat# A21206; RRID:AB_2535792
Donkey anti-rabbit Alexa Fluor 568	Thermo Fisher	Cat# A10042; RRID:AB_2534017
Donkey anti-rabbit Alexa Fluor 594	Thermo Fisher	Cat# A21207; RRID:AB_141637
Donkey anti-rabbit Alexa Fluor 647	Thermo Fisher	Cat# A31573; RRID:AB_2536183
Donkey anti-goat Alexa Fluor 488	Thermo Fisher	Cat# A11055; RRID:AB_2534102
Donkey anti-goat Alexa Fluor 594	Thermo Fisher	Cat# A11058; RRID:AB_2534105
Donkey anti-mouse IRDye®680RD	Licor	Cat# 926-68072; RRID:AB_10953628
Donkey anti-mouse IRDye®800CW	Licor	Cat# 926-32212; RRID:AB_621847
Donkey anti-rabbit IRDye®680RD	Licor	Cat# 926-68073; RRID:AB_10954442
Donkey anti-rabbit IRDye®800CW	Licor	Cat# 926-32213; RRID:AB_621848
Donkey anti-goat IRDye®680RD	Licor	Cat# 925-68074; RRID:AB_2650427
Donkey anti-goat IRDye®800CW	Licor	Cat# 926-32214; RRID:AB_621846
Donkey anti-sheep IgG conjugated to colloidal gold 6 nm	Aurion	Cat# 806.344
Goat anti-rabbit IgG conjugated to colloidal gold 5nm	Aurion	Cat# 806.011; RRID:AB_2732799

**Bacterial and virus strains**		

IAV strain A/Puerto Rico/8/34	Gift from PD	PR8
IAV strain A/Puerto Rico/8/34 with NS1 mutant	Gift from PD	NS1-N81
HSV-1 wild-type	ECS lab	17+

**Chemicals, peptides, and recombinant proteins**		

DMEM	Sigma-Aldrich	Cat# D5796
DMEM, phenol red free, 25mM HEPES	Gibco	Cat# 21063-029
Fetal Bovine Serum	Gibco	Cat# 10270
Sodium Pyruvate	Gibco	Cat# 11360-039
Penicillin Streptomycin	Gibco	Cat# 15140-122
OPTI-MEM	Gibco	Cat# 31985-062
Lipofectamine2000	Thermo Fisher	Cat# 11668027
Geneticin	Gibco	Cat# 10131-019
Doxycycline hydrochloride	Clontech	Cat# 631311
Poly(I:C)	Sigma-Aldrich	Cat# P1530
TPCK-treated trypsin	Worthington	Cat# LS003740
BSA, fraction V	Sigma-Aldrich	Cat# A-7906
Avicel RC-591	Dupont Pharma	Cat# RC-591
Formaldehyde 37%	Honeywell	Cat# F1635
Toluidine blue	Sigma-Aldrich	Cat# 89640
Triton X-100	Sigma-Aldrich	Cat# X100
DAPI	Biotium	Cat# 40043
Fluoromount G	Invitrogen	Cat# 00-4959-52
Vectashield	Vector	Cat# H1000
Glycine	Sigma-Aldrich	Cat# G8790
Glutaraldehyde 10%	Electron Microscopy Services	Cat# 16120
Sucrose	Sigma-Aldrich	Cat# 84097
Uranyl acetate	Agar Scientific	Cat# R1260A
TransIT-X2 transfection reagent	Mirus Bio	Cat# MIR6003
AEBSF	Sigma-Aldrich	Cat# SBR00015
PMSF	Merck	Cat# 329-98-6
Aprotinin	Sigma-Aldrich	Cat# A1250000
Pepstatin A	ThermoScientific	Cat# 78436
Leupeptin hemisulfate	Sigma-Aldrich	Cat# L8511
RNase A	Qiagen	Cat# 1007885
DNAse I	Invitrogen	Cat# 18047-019
Trypsin, proteomics grade	Sigma-Aldrich	Cat# T7575
jetPrime transfection reagent	Polyplus	Cat# 114-15
Fugene 6 transfection reagent	Promega	Cat# E2693
Superscript II reverse transcriptase	Invitrogen	Cat# 100004925

**Critical commercial assays**		

Dual-Glo® Luciferase Assay System	Promega	Cat# E2920
RNeasy mini kit	Qiagen	Cat# 74104

**Deposited data**		

Table S1	This paper	Mendeley Data doi: https://doi.org/10.17632/xgwb62nznh.1

**Experimental models: Cell lines**		

Human: HT1080	ATCC	CCL-121 (RRID:CVCL_0317)
Human: HEK293FT	ThermoFisher	R700-07 (RRID:CVCL_6911)
Human: HEK293T	ATCC	CRL-3216 (RRID:CVCL_0063)
*Canis lupus familiaris*: MDCK	ATCC	CCL-34 (RRID:CVCL_0422)

**Oligonucleotides**		

siRNA CONTROL Sense – AAUUCUCCG AACGUGUCACGU[dT][dT] Antisense - ACGUGACACGUUCGGAG AAUU[dT][dT]	Sigma-Aldrich	N/A
siRNA SYNCRIP Sense - CUAUCGUGG UGGAUAUGAAGA[dT][dT] Antisense - UCUUCAUAUCCACCACG AUAG[dT][dT]	Sigma-Aldrich	N/A
siRNA MEN1 Sense - GAUCAUGCCUG GGUAGUGUU[dU][dG] Antisense - AACACAUACCCAGGCAU GAUC[dC][dU]	Sigma-Aldrich	N/A
siRNA DDX5 Sense – CCCAAUAAGA CUUUAGAAGUA[dT][dT] Antisense - UACUUCUAAAGUCUUA UUGGG [dT][dT]	Sigma-Aldrich	N/A
siRNA SNRNP70 Sense – GGUCUACA GUAAGCGGUCA[dT][dT] Antisense - UGACCGCUUACUGUAG ACC [dT][dT]	Sigma-Aldrich	NA
siRNA RPS27A Sense – UUAGUCGCCUU CGUCGAGA[dT][dT] Antisense - UCUCGACGAAGGCGAC UAA [dT][dT]	Sigma-Aldrich	N/A
siRNA TCERG1 Sense – GGAGUUGCACA AGAUAGUU[dT][dT] Antisense – AACUAUCUUGUGCAACU CC[dT][dT]	Sigma-Aldrich	N/A
siRNA AATF Sense – AAGCGCUCUGCC UACCGAGUU[dT][dT} Antisense – AACUCGGUAGGCAGAGC GCUU[dT][dT]	Sigma-Aldrich	N/A
siRNA STING Sense#1 – GCACCUGUG UCCUGGAGUA[dT][dT] Antisense#1 – UACUCCAGGACACAG GUGC[dT][dT]	Sigma-Aldrich	N/A
siRNA STING Sense#2 – GCAUCAAGG AUCGGGUUUA[dT][dT] Antisense#2 – UAAACCCGAUCCUUG AUGC[dT][dT]	Sigma-Aldrich	N/A
qPCR Primer: IFNβ Fw 5’-CCTGAAGG CCAAGGAGTACA-3’ and Rev 5’-AGCAATTGTCCAGTCCCAGA-3’	Integrated DNA Technologies	N/A
qPCR Primer: GAPDH Fw 5’-GTGAAG GTCGGAGTCAACG-3’ and Rev 5’ATGACAAGCTTCCCGTTCTC-3’	Integrated DNA Technologies	N/A

**Recombinant DNA**		

pLVX-TRE3G	Clontech	Cat# 631358
pcDNA3.1	ThermoFisher	Cat# V79020
LaminA-GFP	ECS lab	Cat# 473
RFP-GFP	ECS lab	Cat# 550
STING-RFP	ECS lab	Cat# 446
STING-GFP	ECS lab	Cat# 596
Dox-inducible STING-GFP	ECS lab	Cat# 1767
NET55-GFP	ECS lab	Cat# 610
Luciferase reporter construct: IFNbeta-firefly	Gift from GJT	N/A
Luciferase reporter construct: NFKB-firefly	Gift from GJT	N/A
Luciferase reporter construct: pRL-TK-Renilla	Promega	Cat# E2241
cGAS plasmid, pCDNA-FLAG-cGAS	Gift from GJT	N/A

**Software and algorithms**		

ImageJ, Fiji	NIH, Schneider et al., 2012	Version: 2.1.0 (RRID:SCR_00228)
GDSC SMLM Fiji plugin	Herbert, 2013	http://www.sussex.ac.uk/gdsc/intranet/microscopy/UserSupport/AnalysisProtocol/imagej/gdsc_plugins/
BioConductor, GOstats	Falcon and Gentleman, 2007	https://bioconductor.org/packages/release/bioc/html/GOstats.html (RRID:SCR_008535)
FLIMfit 5.1.1	Imperial College London	https://flimfit.org/
Macros for FRAP analysis	DAK, COIL, University of Edinburgh	https://coil.bio.ed.ac.uk

## Data Availability

• Raw and processed proteomics datasets have been deposited in Mendeley Data with doi: https://doi.org/10.17632/xgwb62nznh.1 listed in the key resources table. All other data reported in this paper will be shared by the lead contact upon request. • The macros used to correct and analyze FRAP data will be made available upon request to the Wellcome Centre for Cell Biology Centre Optical Imaging Laboratory at https://coil.bio.ed.ac.uk. • Any additional information required to reanalyze the data reported in this paper is available from the lead contact upon request.

## Supplemental information

Document S1. Figures S1–S4

Table S1. STING NE partners identified by tandem mass spectrometry, related to Figure 3 List of proteins identified by mass spectrometry in STING-GFP and mock-transfected control pulldown samples. STING-GFP enriched ‘top hits’ are highlighted in orange and show proteins that have a normalised spectral score at least 2-fold enriched over the mock sample and are represented by 2 or more peptides or by 1 peptide with 2 or more spectra.

## STAR★ Methods

### Resource availability

#### Lead contact

The lead contact for this study is prof Eric C Schirmer. All information and requests for resources should be directed to and will be fulfilled by the prof. Schirmer e.schirmer@ed.ac.uk.

#### Materials availability

• Plasmids generated for this study will be deposited in Addgene.
• Stable cell lines will be available upon request.
• No other unique reagents were generated for this research.

### Experimental model and subject details

#### Cells

HT1080, MDCK, HEK293T and HEK293FT cells were maintained in high glucose DMEM (Sigma) supplemented with 10% fetal bovine serum (FBS), 100 μg/μl penicillin and 100 μg/μl streptomycin sulfate (Gibco). To generate a stable inducible NET23/STING expressing cell line, a lentivirus vector encoding doxycycline inducible NET23/ STING fused to GFP at the C-terminus (pLVX-TRE3G backbone, Clontech) was prepared by standard procedures and transduced into HT1080 cells. Transduced cells were selected with geneticin at 500 μg/ml (Gibco). Inducible stable cells were treated with 0.05 μg/ml doxycycline (Clontech) for 20 h in order to induce NET23/STING expression before use in experiments.

#### Generation of viral stocks, virus titrations, and infections

IAV strain A/Puerto Rico/8/34 (PR8) stocks were generated using reverse genetics as previously described (de Wit et al., 2004; Pereira et al., 2017). 8 pDUAL plasmids (250 ng each with 4 μl Lipofectamine 2000 (Life Technologies, Loughborough, UK) containing sequences of a complete IAV genome were used to transfect HEK293T cells simultaneously, with transfections either incorporating the WT segment 8 sequence or an NS1 mutant, NS1-N81 (Pereira et al., 2017). After overnight incubation, virus growth medium (DMEM supplemented with 5 μg/ml TPCK-treated trypsin, 0.14% BSA fraction V and penicillin (100 U/ml) and streptomycin (100 μg/ml)) was added to allow a small-scale amplification of the viruses in HEK293T cells. After 48 h, the virus particle-containing supernatants were passaged on MDCK cells to further amplify the viruses to obtain working stocks.

Virus titres were determined by plaque titration on MDCK cells in 6 well plates. Cells were inoculated with virus dilutions for 1 h, then an overlay (mixture of equal volume of DMEM and 2.4% Avicel (Sigma-Aldrich, UK) supplemented with 1 μg/ml TPCK-treated trypsin and 0.14% BSA fraction V) was put onto the wells. After 48 h, cells were fixed using 3.7% formaldehyde and stained with 0.01% Toluidine Blue. Virus titres were calculated by plaque count∗dilution factor/(volume of inoculum) and expressed as PFU/ml.

HT1080 cells were mock infected or infected with either wild-type PR8 or NS1-N81 mutant virus at an MOI of 0.01 or 3 as indicated in figure legends. Culture media of infected cells was collected at time points given post-infection for use in plaque assays, or cells were harvested in Laemmli buffer for Western blotting.

HSV-1 wild type strain 17+ viral stocks were generated in BHK-21 cells. Plaque assays were used to determine stock viral titre using Vero cells seeded in a 12-well plate. On the day of the assay 90% confluent cells were infected with 10-fold serial dilutions of HSV-1 diluted in serum-free medium (10^-1^ to 10^-8^). Cell growth medium was removed from wells and 100 μL of virus dilution was added per well, with each virus dilution used to infect cells in duplicate. Plates were incubated for 1 h at 37°C, gently rocking the plate every 15 min. After 1h, virus inoculum was removed, and cells were overlaid with 2 mL of overlay medium (1:1 mix of 2X MEM growth medium and 1.2% Avicell (RC-591 NF) diluted in distilled water and autoclaved). Plates were incubated for 3 days at 37°C before fixation and staining. Overlay medium was removed and cells were fixed for 30 min in 3.7% formaldehyde. Fixative was then removed, and cells were stained with 2% crystal violet solution. Quantification of viral titres was determined by counting the number of plaques in a well with a dilution that gave between 10 and 100 plaques and viral titre (PFU/mL) was calculated. Cells were infected with HSV-1 at an MOI of 10 for use in experiments.

### Method details

#### IIR induction

To induce IIR in 6-well plates for immunoblotting and qPCR experiments, cells were transfected with 10 μg poly(I:C) (Sigma), 5 μg pcDNA3.1, or transfection reagent alone (mock) using 4 μl Lipofectamine 2000 transfection reagent in 200 μl optiMEM added to cells in 2 ml culture medium. For immunofluorescence staining to quantify nuclear IRF3/NFκB, cells plated onto glass coverslips in 24-well plates were transfected with 2 μg poly(I:C) (Sigma), 1 μg pcDNA3.1, or transfection reagent alone (mock) using 1 μl Lipofectamine 2000 transfection reagent in 50 μl optiMEM added to cells in 0.5 ml culture medium.

#### Standard immunofluorescence microscopy

HT1080 cells seeded on glass coverslips were fixed in 3.7% formaldehyde for 15 min. Cells were then washed in PBS and permeabilized with 0.2% Triton X-100 for 7 min before blocking in 4% BSA for 30 min at room temperature. Cells were incubated with primary antibodies in blocking buffer overnight at 4°C, antibodies used were: anti-STING (AF6516, R&D Systems), anti-Nup153 (QE5, ab24700, Abcam), anti-IRF3 (FL-425, sc-9082, Santa Cruz Biotechnology), anti-NFκB (L8F6, 6956S, Cell Signaling Technology). The following day, coverslips were washed with PBS and incubated for 1 h with appropriate secondary antibodies conjugated to Alexa Fluor dyes (MolecularProbes). DNA was visualized with DAPI (4,6-diamidino-2 phenylindole, dihydrochloride) and coverslips mounted in fluoromount G (EM Sciences). A second anti-STING antibody was also used (D2P2F, #13647, Cell Signaling Technology) that required an alternative fixation in pre-chilled methanol for 15 min at -20°C. Immunofluorescent staining was then carried out as described in (Almine et al., 2017). Images were obtained using a Zeiss AxioImager equipped with 1.45 NA 100x objective.

#### Structured illumination microscopy

HEK293T cells transfected with STING-GFP were fixed for 7 min in 3.7% formaldehyde, washed with PBS, and then permeabilized 6 min with 0.2% Triton X-100 in PBS. Cells were then blocked with 10% FBS, 200 mM glycine in PBS, and reacted for 40 min at RT with antibodies to Nup153 (Covance) or Nup358 (kind gift of F. Melchior). All secondary antibodies were Alexa fluor highly immunoadsorbed goat IgG (MolecularProbes) matched to the OMX system used. DNA was visualized with DAPI and coverslips mounted in Vectashield.

Structured illumination images (Figure 1) were taken on the OMX system at the University of Dundee microscopy facility (details described at http://microscopy.lifesci.dundee.ac.uk/omx/). OMX was employed to determine whether a protein is in the ONM or the INM by co-staining with antibodies to nuclear pore complex (NPC) cytoplasmic filament protein Nup358 and nuclear basket protein Nup153 as these structures extend well beyond the 50 nm spacing between the inner and outer nuclear membranes.

#### Immunogold electron microscopy

HT1080 control cells or HT1080 cells stably transfected with inducible STING-GFP that was induced O/N with doxycycline were fixed for 2 min by the addition of 2X fixation buffer (8% formaldehyde, 0.2 M PHEM buffer (120 mM PIPES, 20 mM EGTA, 50 mM HEPES, 4 mM MgCl_2_, pH 6.9)) to culture medium, this was then replaced with 1X fixation buffer (4% formaldehyde, 0.1 M PHEM buffer, 0.05% glutaraldehyde) and fixation continued for 2 h. Cells were then scraped and transferred to microfuge tubes, before pelleting and washing with PBS. Cell pellets were then stored in PBS prior to sectioning and immunostaining. The cell pellets were embedded in 10% gelatin before being infused overnight with 15% PVP and 1.7 M sucrose in 0.1 M phosphate buffer. Liquid nitrogen frozen pellets were sectioned on a cryo-ultramicrotome (UC7 with FC7 cryo-attachment; Leica). Immunoelectron microscopy was performed using the Tokuyasu method (Tokuyasu, 1973). Cryosections were thawed, rinsed with 1% glycine in PBS with and blocked with 1% BSA in PBS. For endogenous STING, grids were incubated with sheep anti-STING antibody at 1:7 dilution (AF6516, R&D Systems), rinsed in PBS, then incubated with donkey anti–sheep IgG conjugated to 6 nm colloidal gold (Aurion). For STING-GFP expressing cells grids were incubated with rabbit anti-GFP (Abcam) at 1:20 dilution, rinsed in PBS, then incubated with goat anti–rabbit IgG conjugated to 5 nm colloidal gold (Aurion). Grids were then rinsed in PBS, transferred to 1% glutaraldehyde (Agar Scientific) in PBS, washed in water, and embedded in 2% methyl cellulose containing 0.4% uranyl acetate (Agar Scientific). Imaging was performed at 100 kV with a Hitachi H7600 TEM and Xarosa 20 Megapixel camera.

#### FRET-FLIM

HEK293T cells were seeded at 70,000 cells per well on No. 1.5 glass coverslips in 24-well plates. The next day cells were transiently transfected with plasmids encoding Lamin A-GFP, an RFP-GFP tandem construct, or Lamin A-GFP and STING-RFP (Malik et al., 2010). After 24 h cells were fixed 15 min in 3.7% formaldehyde before washing in PBS and mounting in Vectashield®. Imaging was performed on a Leica SP5 SMD confocal laser scanning microscope equipped with PicoHarp 300 (TCSPC module and picosecond event timer) and single photon avalanche detectors. Leica application suite with FLIM wizard software and integrated Symphotime software were used for single photon counting acquisition and FLIM measurements carried out for 5 min per field of view. FLIM data was analyzed using FLIMfit 5.1.1 software (the FLIMfit software tool developed at Imperial College London).

#### FRAP

For FRAP experiments, HT1080 cell lines stably transfected with doxycycline-inducible STING-GFP or NET55-GFP constructs were plated onto 25 mm round coverslips in 6-well plates (200,000 cells/well). These were first treated 20 h prior to IIR induction with 0.05 μg/ml doxycycline to induce STING or NET55 expression. For stimulation of IIR cells were subsequently infected with a multiplicity of infection (MOI) of 10 using HSV-1 strain 17+. The virus was added to cells in 0.5 ml culture medium. The cells were incubated for 1 h at 37°C and 5% CO_2_ to facilitate adsorption of the virus. Subsequently 1.5 ml medium was added and the cells were incubated for an additional 2 h before FRAP imaging. For poly(I:C) stimulation, similarly prepared and induced cells in 6-well plates were transfected with 10 μg poly(I:C) using Lipofectamine 2000 (Invitrogen) for 2 h before imaging. All fluorescence recovery after photobleaching (FRAP) experiments for GFP-fusion constructs were performed on a Leica SP5 microscope equipped with an Argon laser using the 488 nm laser line and a 60x HCX PLAPO NA 1.4 oil objective. At the stated times after IIR induction, coverslips were individually removed from the 6-well dish and clamped into an environmental chamber (Life Imaging Services, Switzerland) with 2 ml of preheated (37°C), phenol-free complete DMEM containing 25 mM Hepes-KOH. Temperature was maintained at 37°C in the chamber and cells were gassed using 5% CO_2_ in air using a gas mixer (Life Imaging Services). Five pre-bleach images were taken followed by bleaching a spot of 1 μm for 1 s at full laser intensity. Subsequent images were taken in 2 phases. The first rapid phase consisted of 25 frames every 0.65 s to observe the rapid initial recovery. The second slow phase consisted of 75 frames every 2 s to observe the slower final stages of recovery, these parameters were shown by preliminary experiments (data not shown) to allow complete steady state recovery in all cells.

Image data was processed using Image-Pro Premier (Media Cybernetics Inc., MD, USA). Background and photobleach corrections were engaged using an algorithm written by D.A.K. according to (Phair and Misteli, 2001). For subsequent FRAP measurements, a macro was written by D.A.K. in VB.Net within Image Pro Premier whereby a region of interest (ROI) was applied to the bleach spot, background and non-bleached area of a nearby cell and corrected for movement automatically compared to the 5 pre-bleach images. The t½s were calculated from the normalized fluorescence values.

#### Single-Molecule Fluorescence Recovery After Photobleaching (smFRAP) microscopy

Imaging was performed using an Olympus IX81 equipped with a 1.4-NA 100x oil-immersion apochromatic objective (UPLSAPO 100XO, Olympus, Center Valley, PA). An Obis^TM^ solid state 488-nm (Coherent Inc, Santa Clara, Ca) was passed through dichroic filters (Di01- R405/488/561/635-25x36, Semrock, Rochester, NY) and emission filters (NF01- 405/488/561/635-25X5.0, Semrock, Rochester, NY) as well a circular variable metallic neutral density filter (Newport, Irvine, CA) and directed into the microscope using a micrometer stage (Newport, Irving, CA). Cells modified to express doxycycline inducible STING-GFP were plated on No. 0 cover glass 35 mm petri dishes with 14 mm Microwell (MatTek, Ashland, MA). Cells were transfected via lipofection agent Transit-X2 (Mirus Bio LLC, Madison, WI) 2 h prior to imaging for plasmid DNA or poly(I:C), or transfection reagent alone (mock). Growth media was replaced with Transport Buffer (20 mM HEPES, 110 mM KOAc, 5 mM NaOAc, 2 mM MgOAc, and 1 mM EGTA, pH 7.3) 30 min prior to imaging to slow membrane movements and reduce autofluorescence. Imaging was performed on the NE region opposite the ER. The region was photobleached for 1 min at 5 mW and then imaged for 30 s at 50 μW. An optical chopper rotating at 2-Hz was utilized during capture to allow for fluorescence recovery. Images were captured using the Slidebook software package (Intelligent Imaging Innovation, Denver, Co). Data were analyzed using the FIJI ImageJ (Schindelin et al., 2012; Schneider et al., 2012) plugin GDSC SMLM (Single Molecule Light Microscopy ImageJ Plugins, University of Sussex, http://www.sussex.ac.uk/gdsc/intranet/microscopy/UserSupport/AnalysisProtocol/imagej/gdsc_plugins/;(Herbert, 2013)) and OriginPro 2019 (OriginLab, Northampton, MA) (Tingey et al., 2021).

#### Nuclear-specific cross-linking co-IP

2 x 10^8^ HEK293T cells were transfected using Ca_2_PO_4_ with either STING-GFP or mock transfected. As overexpression of STING tends to eventually result in apoptosis, cells were taken at ∼20 h post-transfection. Note that a mock-transfected cell population was prepared for reversible cross-link immunoprecipitations the same way as the transfected cells. Cells were first rinsed on the plates with PBS, then incubated with trypsin for 3 min and shaken from the plates. Recovered cells were washed in 10% FBS in PBS to inactivate trypsin and pelleted by centrifugation at 250 x g for 10 min at room temperature. The NE isolation followed published protocols (Korfali et al., 2009, 2010). For all subsequent steps the following protease inhibitors were added freshly to solutions: 1 mM AEBSF [4-(2-Aminoethyl) benzenesulfonyl fluoride hydrochloride], 1 μg/ml aprotinin, 1 μM pepstatin A, and 10 μM leupeptin hemisulfate. Cells were then washed with PBS, repelleted and next resuspended in roughly 100x the pellet volume with hypotonic lysis buffer (10 mM HEPES pH 7.4, 1.5 mM MgCl_2_, 10 mM KCl with freshly added 2 mM DTT and protease inhibitors). Cells were allowed to swell on ice for 5 min and then nuclei were released by dounce homogenization with 10 vigorous strokes using a type B pestle (Wheaton, clearance between 0.1 and 0.15 mm). Thereupon 1/10 volume of 1 M KCl and 1/10 volume of 2.2 M sucrose were immediately added. Nuclei were pelleted through a 0.9 M sucrose cushion in the same buffer with the salt (0.9 M sucrose, 10 mM HEPES pH 7.4, 1.5 mM MgCl_2_, 110 mM KCl with freshly added DTT) at 2,000 x g in a swinging bucket rotor (e.g. 4,000 rpm in a Beckman Coulter J6-MC floor model centrifuge) for 20 min at 4°C. Nuclear pellets were resuspended at 1.5 million nuclei/ ml in 0.25 M sucrose, 10 mM HEPES pH7.4, 10 mM KCl, 2 mM MgCl_2_, 1.5 mM CaCl_2_, with 2 mM DTT, protease inhibitors and 4 U/ml DNase and 1 μg/ml RNase and incubated at room temperature to digest chromatin while swelling nuclei to release the digested chromatin. These were then pelleted through a 0.9 M sucrose cushion as before except at 6,000 x g using a swinging bucket rotor for 20 min at 4°C. The process was repeated with increasing the DNase to 20 U/ml and the RNase to 10 μg/ml and pelleted as above to generate a crude NE fraction, though this time the pellet was resuspended in the same buffer lacking the DNase, RNase, and DTT.

During the DNase/RNase incubations the *ortho*-phenanthroline copper was prepared by mixing a solution of 200 mM CuSO_4_ in water at 1:1 with 400 mM 1,10-*ortho*-phenanthroline in ethanol. The cross-linker complex is allowed to form by gentle rotation for 30 min at room temperature. The *ortho*-phenanthroline copper solution was then added at 1:100 to the NEs resuspended without DTT and incubated for 30 min at 30°C. The reaction was stopped by addition of EDTA to 5 mM and the cross-linked NEs were pelleted at 6,000 x g using a swinging bucket rotor for 20 min at 4°C.

The pellet was resuspended in RIPA buffer with freshly added protease inhibitors and incubated for 20 min at room temperature. This material was then sonicated on ice for 5 min using a bath sonicator with a rotating cycle of 15 s on and 15 s off. Large insoluble material was removed by pelleting at 100 x g for 1 min and antibodies were added for immunoprecipitations. Both mock transfected lysate and STING-GFP transfected lysate were separately incubated with antibodies against GFP (Life Technologies A11122). All antibodies were incubated overnight at 4°C with gentle rotation. The next day antibody-complex conjugates were incubated with Protein A-sepharose beads and washed on the beads with 0.5x RIPA buffer with freshly added protease inhibitors 3 times. Complexed proteins were then released from the beads with 50 mM DTT incubated at room temperature for 30 min and material processed for mass spectrometry.

#### SDS-PAGE analysis and in-gel digestion for mass spectrometry

Released proteins were in-gel digested as described elsewhere (Shevchenko et al., 1996). In brief, a band of coomassie-stained gel was excised and the proteins where digested using trypsin and proteins were reduced in 10 mM DTT for 30 min at 37°C, alkylated in 55 mM iodoacetamide for 20 min at room temperature in the dark, and digested overnight at 37°C with 12.5 ng/μL trypsin (Proteomics Grade, Sigma). The digestion media was then acidified to 0.1% of TFA and spun onto StageTips as described in the literature (Rappsilber et al., 2003). Peptides were eluted in 20 μL of 80% acetonitrile in 0.1% TFA and were concentrated to 4 μL (Concentrator 5301, Eppendorf AG). The peptides sample was then diluted to 5 μL by 0.1% TFA for LC-MS/MS analysis.

#### Mass spectrometry analysis

An LTQ-Orbitrap mass spectrometer (Thermofisher Scientific) was coupled on-line to an Agilent 1100 binary nanopump and an HTC PAL autosampler (CTC). The peptides were separated using an analytical column with a self-assembled particle frit (Ishihama et al., 2002) and C_18_ material (ReproSil-Pur C18-AQ 3 μm; Dr. Maisch, GmbH) was packed into a spray emitter (100-μm ID, 8-μm opening, 80-mm length; New Objective) using an air-pressure pump (Proxeon Biosystems). Mobile phase A consisted of water, 5% acetonitrile, and 0.5% acetic acid; mobile phase B, consisted of acetonitrile and 0.5% acetic acid. The gradient used was 98 min. The peptides were loaded onto the column at a flow rate of 0.7 μL/min and eluted at a flow rate of 0.3 μL/min according to the gradient. 0% to 5%B in 5 min, 5% to 20% buffer B in 80 min and then to 80% B in 13 min. FTMS spectra were recorded at 30,000 resolution and the six most intense peaks of the MS scan were selected in the ion trap for MS2, (normal scan, wideband activation, filling 7.5E5 ions for MS scan, 1.5E4 ions for MS2, maximum fill time 150 ms, dynamic exclusion for 150 s).

Searches were conducted using Mascot software (Version 2.2.0) against a database containing Human sequences (sprothuman201106). The search parameters were: MS accuracy, 6 ppm; MS/MS accuracy, 0.6 Da; enzyme, trypsin; allowed number of missed cleavages, 2; fixed modification, carbamidomethylation on Cysteine; variable modification, oxidation on Methionine.

#### siRNA knockdowns

For HT1080 and HEK293FT cells, one day prior to knockdown cells were seeded at 300,000 cells/well of a 6-well plate. The next day siRNA knockdowns were performed using siRNA duplexes listed in the key resources table and jetPRIME transfection reagent (POLYPLUS) according to the manufacturer’s instructions for a final concentration of 50 nM siRNA, 4 μl jetPRIME, 200 μl buffer per well. For STING knockdown a mix of siRNA#1 and #2 were used for a final concentration of 50 nM. 24 h later cells were trypsinized and re-seeded 1:2 in 6 well plates for western blotting and qPCR experiments, or at 50,000 cells/well of a 24-well plate on 13 mm No. 1.5 glass coverslips for immunofluorescence.

#### Dual-luciferase reporter assay

Following siRNA knockdown transfection in 6-well plates as described for HT1080 cells, 5x10^4^ cells were seeded per well of a 48-well plate. After 24 h, cells were co-transfected with plasmids expressing luciferase reporter constructs (IFNβ-firefly or NF-κB-firefly at 30 ng and Prl-TK-renilla at 5 ng) and either cGAS and STING (20ng each) or empty vector DNA (pcDNA3.1 at 40 ng). For each well, 1 μL of Fugene (Promega) was incubated with 25 μL of Opti-MEM for 5 min at RT before the addition of plasmid constructs and 280 ng of the required siRNA oligos (second knockdown). This final mixture was incubated for a further 20 min at RT before addition to each well containing 150 μL fresh media. After 24 h media was exchanged for 300 μL fresh media.

To measure luminescence produced by luciferase activity, cells were harvested 96 h after the initial siRNA knockdown transfection. Media was removed and cells were washed in PBS before re-suspension in 75 μL 1X Passive Lysis Buffer (PLB; Dual-Luciferase Reporter Kit, Promega). Cells were incubated in PLB for 15 min at RT and further homogenized by pipetting. 25 μL of the homogenized lysate was transferred to a well on a 96-well plate and luminescence signal detected using a Modulus Microplate Multimode Reader (Turner Biosystems). The plate reader was programmed to inject 40 μl of Luciferase Assay Substrate re-suspended in Luciferase Assay Buffer II and 35 μl of 1X Stop&Glo Reagent (Dual-Luciferase Reporter Kit, Promega) per well, with a 2 s delay between injections and 10 s measurement period after each injection. Luminescence signal produced by IFNβ/NF-κB-firefly luciferase was divided by pRL-TK-renilla luciferase luminescence signal to control for variation in transfection efficiency and cell number.

#### qPCR

RNA extraction was achieved using RNeasy Mini Kit (Qiagen) as per the manufacturer’s instructions. The quality and concentration of the RNA was then assessed using a NanoDrop 2000c spectrophotometer and run on a TAE gel to check for degradation. If not immediately used, RNA was stored at -80°C in separate aliquots to reduce freeze thawing. RNA was converted into cDNA using SuperScript II reverse transcription reagent (Invitrogen) and quantitative real time-PCR (qRT-PCR) performed with normalization to GAPDH. The cycle program consisted of an initial denaturing step of 5 min at 95°C, then 45 cycles of 10 s at 95°C, 15 s at 51°C, and 20 s at 72°C, followed by a melting curve analysis step. qPCR primers were: IFNβ Fw5’-CCTGAAGGCCAAGGAGTACA-3’ and Rev 5’-AGCAATTGTCCAGTCCCAGA-3’ and GAPDH Fw 5’-GTGAAGGTCGGAGTCAACG-3’ and Rev 5’ATGACAAGCTTCCCGTTCTC-3’.

#### Immunoblotting

For immunoblotting cell lysates were collected directly by addition of 2x sample buffer (with the addition of Na_3_VO_4_ and NaF for phosphorylated proteins) to washed cell monolayers. Samples were boiled for 5 min at 95°C and sonicated 1 s on 1 s off with a probe sonicator before separation of proteins by SDS-PAGE. Proteins were transferred to nitrocellulose membranes, blocked with 5% BSA or Milk for 1 h at room temperature before probing with primary antibodies overnight. Primary antibodies were: anti-STING (AF6516, R&D Systems), anti-STING (D2P2F, #13647, Cell Signaling Technology) anti-IRF3 (FL-425, sc-9082, Santa Cruz Biotechnology), anti-cGAS (HPA031700, Atlas Antibodies), anti-cGAS (D1D3G, #15102, Cell Signaling Technology), anti-phospho-IRF3 (S386) (ab76493, Abcam), anti-H3 (ab10799, Abcam), anti-gamma tubulin (GTU-88, T6557, Merck), anti-alpha tubulin (ATN02, Cytoskeleton Inc.), anti-SYNCRIP (7A11.2, MAB11004, Millipore), anti-MEN1 (ab2605, Abcam), anti-DDX5 (ab10261, Abcam), anti-snRNP70 (ab51266, Abcam), anti-RPS27a (ab172293, Abcam), anti-AATF (ab39631, Abcam), anti-Lamin A/C (3262, generated by ECS), anti-NS1 (NS1-RBD, generated by PD), anti-NP (A2915, generated by PD), anti-Calnexin (SPA-860, Stressgen).

#### Cellular fractionation of nuclei and microsomal membranes

Adherent cells grown on 60-mm dishes were trypsinized and cell pellets washed with ice-cold PBS. Cell pellets were resuspended in 0.7 mL ice-cold hypotonic lysis buffer (20 mM Tris-HCl (pH 7.4), 10 mM KCl, 2mM MgCl_2_, 1 mM EDTA, 0.5 mM DTT, 0.5 mM PMSF) and allowed to swell for 10 min on ice. Nuclei were released from cells using a 15 mL Dounce homogeniser, with 40 vigorous strokes sufficient to release most nuclei, as determined by microscopic analysis. Immediately after Dounce homogenization 70 μL of 1 M KCl was added to prevent osmotic rupture of nuclei. Nuclei were pelleted by centrifugation at 1000 x g at 4°C for 3 min. Pelleted nuclei were resuspend in 2x sample buffer while supernatant containing cytoplasm and other cell membranes was subject to ultracentrifugation at 104,000 x g at 4°C for 25 min to pellet the microsomal membrane fraction. After centrifugation supernatant was discarded and the microsomal membrane pellet resuspended in 2x sample buffer. Nuclear and microsomal membrane fractions were then run on SDS-PAGE and probed for STING levels by Western blot.

#### Bioinformatics analysis

Gene ontology (GO) analysis was performed in R using the Bioconductor package GOstats (Falcon and Gentleman, 2007). Significantly over-represented categories and other biologically interesting classes were represented as a piechart. Only experimentally verified GO-functional annotations were used, including EXP (Inferred from EXPeriment), IDA (Inferred from Direct Assay), and IPI (Inferred from Physical Interaction). We used the following GO terms: “defense response” (GO:0006952), “immune response” (GO:0006955), “response to virus” (GO:0009615), “apoptosis” (GO:0006915), “cell cycle” (GO:0007049), “regulation of cell cycle” (GO:0051726), “negative regulation of cell proliferation” (GO:0008285), “positive regulation of cell proliferation” (GO:0008284), “regulation of transcription” (GO:0006355), “transcription factor” (GO:0001071), “RNA splicing” (GO:0008380), “RNA binding” (GO:0003723), “DNA binding” (GO:0003677), “chromatin” (GO:0000785), “chromatin binding” (GO:0003682), “chromatin modification” (GO:0016568), “chromatin organization” (GO:0051276). To plot MS data piecharts, the relative proportions of the various classes were calculated from the relative abundances of the genes identified from each GO category, and this was compared to a piechart compiled from all the genes in the human genome considering the same categories. The heatmaps simply highlight which genes have been annotated with each GO term shown.

To map further interactions of proteins co-immunoprecipitated with STING, the proteins were searched against the human protein reference database (HPRD, Johns Hopkins University) to identify primary and secondary interactors, using Cytoscape (Shannon et al., 2003) for visualization.

### Quantification and statistical analysis

Statistical analyses were performed using Prism (GraphPad Software), statistical tests used, values of n, and measurements performed are detailed in figure legends. For Figure 1E Ordinary one-way ANOVA with Dunnett’s multiple comparison test, ∗∗∗∗ p ≤ 0.0001 was used. For Figure 2C student's T test, ∗p ≤ 0.05, for Figure 2F statistical test used was ordinary one-way ANOVA with Dunnett’s multiple comparisons test, ∗∗ p ≤ 0.01, ∗∗∗∗ p ≤ 0.0001, for Figure 2G statistical test used was ordinary one-way ANOVA with Dunnett’s multiple comparisons test, ∗ p ≤ 0.05. For Figure 4C ordinary one-way ANOVA and Dunnett’s multiple comparisons test was used, ∗∗ p ≤ 0.01, ∗∗∗ p ≤ 0.001. For Figure 4D ordinary one-way ANOVA and Dunnett’s multiple comparisons test was used, ∗∗ p ≤ 0.01, ∗∗∗ p ≤ 0.001, ∗∗∗∗ p ≤ 0.0001. For Figures 5C–5F, ordinary one-way ANOVA with Dunnett’s multiple comparisons test were performed ∗ ≤ 0.05, p ∗∗ p ≤ 0.01, ∗∗∗ p ≤ 0.001, p ∗∗∗∗ ≤ 0.0001. For Figure 6A, ordinary one-way ANOVA with Dunnett’s multiple comparisons test ∗∗ p ≤ 0.01 was performed. For Figure 6B student’s T tests, ∗ p ≤ 0.05 were performed. For Figure 6C student’s T tests, ∗ p ≤ 0.05 were performed. For Figure 6E, student’s T tests, ∗ p ≤ 0.05 were performed.

Quantification of Western blot data was performed using Image Studio 5.2 (Li-Cor Biosciences) with median local background correction. Target signal was normalized to housekeeping proteins detailed in figure legends.
